# CircNR3C2 promotes HRD1-mediated tumor-suppressive effect via sponging miR-513a-3p in triple-negative breast cancer

**DOI:** 10.1186/s12943-021-01321-x

**Published:** 2021-02-02

**Authors:** Ya Fan, Jia Wang, Wen Jin, Yifei Sun, Yuemei Xu, Yipin Wang, Xiubin Liang, Dongming Su

**Affiliations:** 1 Department of Pathology, Nanjing Medical University, Nanjing, Jiangsu China; 2 Department of Breast Surgery, Institute of Breast Disease, The Second Hospital of Dalian Medical University, Dalian, Liaoning China; 3 State Key Laboratory of Reproductive Medicine, Department of Pathology, Nanjing Medical University, Nanjing, Jiangsu China; 4 Center of Pathology and Clinical Laboratory, Sir Run Run Hospital of Nanjing Medical University, Nanjing, Jiangsu China; 5 Department of Pathophysiology, Nanjing Medical University, Nanjing, Jiangsu China; 6 Department of Pathology, The Affiliated Drum Tower Hospital of Nanjing University Medical School, Nanjing, Jiangsu China

**Keywords:** HRD1, Vimentin, CircRNA, Breast cancer, Metastasis

## Abstract

**Background:**

E3 ubiquitin ligase HRD1 (HMG-CoA reductase degradation protein 1, alias synoviolin with SYVN1 as the official gene symbol) was found downregulated and acting as a tumor suppressor in breast cancer, while the exact expression profile of HRD1 in different breast cancer subtypes remains unknown. Recent studies characterized circular RNAs (circRNAs) playing an regulatory role as miRNA sponge in tumor progression, presenting a new viewpoint for the post-transcriptional regulation of cancer-related genes.

**Methods:**

Examination of the expression of HRD1 protein and mRNA was implemented using public microarray/RNA-sequencing datasets and breast cancer tissues/cell lines. Based on public RNA-sequencing results, online databases and enrichment/clustering analyses were used to predict the specific combinations of circRNA/miRNA that potentially govern HRD1 expression. Gain-of-function and rescue experiments in vitro and in vivo were executed to evaluate the suppressive effects of circNR3C2 on breast cancer progression through HRD1-mediated proteasomal degradation of Vimentin, which was identified using immunoblotting, immunoprecipitation, and in vitro ubiquitination assays.

**Results:**

HRD1 is significantly underexpressed in triple-negative breast cancer (TNBC) against other subtypes and has an inverse correlation with Vimentin, inhibiting the proliferation, migration, invasion and EMT (epithelial-mesenchymal transition) process of breast cancer cells via inducing polyubiquitination-mediated proteasomal degradation of Vimentin. CircNR3C2 (hsa_circ_0071127) is also remarkably downregulated in TNBC, negatively correlated with the distant metastasis and lethality of invasive breast carcinoma. Overexpressing circNR3C2 in vitro and in vivo leads to a crucial enhancement of the tumor-suppressive effects of HRD1 through sponging miR-513a-3p.

**Conclusions:**

Collectively, we elucidated a bona fide circNR3C2/miR-513a-3p/HRD1/Vimentin axis that negatively regulates the metastasis of TNBC, suggesting that circNR3C2 and HRD1 can act as potential prognostic biomarkers. Our study may facilitate the development of therapeutic agents targeting circNR3C2 and HRD1 for patients with aggressive breast cancer.

**Supplementary Information:**

The online version contains supplementary material available at 10.1186/s12943-021-01321-x.

## Background

Breast cancer is the most commonly diagnosed cancer and the leading cause of death by cancer among females worldwide [1]. As a type of highly heterogeneous malignancy, human breast cancer could be mainly divided into four molecular subtypes characterized by different gene expression patterns of several biomarkers, including estrogen receptor (ER), progesterone receptor (PR), and human epidermal growth factor receptor 2 (HER2) [2]. Compared to other breast cancer subtypes, triple-negative breast cancer expressing neither ER/PR nor HER2 is apt to have a higher frequency of lung, brain, and distant nodal metastasis, representing the most severe form of this disease [3].

Despite the fact that cancer-related mortality is predominantly due to the metastasis of primary tumor and subsequent recurrence at distant sites [4], metastasis-targeting therapies are still relatively insufficient owing to the intratumor heterogeneity throughout this cascade of dissemination [5]. It has been fairly elucidated that epithelial-mesenchymal transition acts as a prominent step involved in breast cancer metastasis, whereby epithelium-like cancer cells gradually lose polarity and intracellular tight junctions, yet gain mesenchymal phenotypes and stem traits [6, 7]. Vimentin, a major constituent of intermediate filament proteins whose expression is significantly elevated in multiple aggressive cancers including TNBC, participates in EMT process with a series of pro-metastasis effects, e.g. cytoskeleton reprogramming and stemness sustentation [8]. Canonical EMT-governing signaling pathways, such as Wnt/β-catenin, TGF-β, and NF-κB were revealed to transactivate Vimentin expression [9], and post-translational modifications (PTMs) like phosphorylation, acetylation, and ubiquitination could also affect the function and stability of Vimentin [10]. Specifically, recent studies showed that E3 ubiquitin ligases TRIM56 and RNF208 decrease cell migration and invasion of ovarian and breast cancer respectively by inducing polyubiquitination-mediated proteasomal degradation of Vimentin, suggesting that the suppression of Vimentin ameliorates metastasis in aggressive cancers [11, 12]. Moreover, a tumor-suppressive effect of E3 ligase HRD1 on breast cancer was identified in our previous research [13], while the underlying mechanisms of HRD1 inhibiting breast cancer progression still need to be unraveled.

Since the rise of high-throughput sequencing technology, there have been exploding explorations about circRNAs from their generation to molecular functions [14, 15], especially focusing on the regulatory role of circRNAs in tumorigenesis and cancer development [16]. Among them, what’s notable is that circANKS1B (hsa_circ_0007294) was demonstrated as significantly upregulated in TNBC against adjacent normal tissues, contributing to breast cancer invasion and metastasis [17]. However, plenty of aberrantly expressed circRNAs in TNBC, examined with RNA sequencing, are remaining to be investigated. Here, we figured out the detailed expression profile of HRD1 in breast cancer subtypes, deciphering its circRNA-regulated antimetastatic activity via targeting Vimentin.

## Materials and methods

### Cell culture and reagents

MCF-7, T-47D, BT474, MDA-MB-231, BT549 and HEK293 cells were acquired from American Type Culture Collection (ATCC, USA) and maintained under a 37 °C condition of 5% CO_2_ and at least 95% humidity. MDA-MB-231 cells were cultured in L-15 medium (Gibco, USA) while all the other cells in Dulbecco’s Modified Eagle Medium (DMEM, Gibco), both with 10% fetal bovine serum (FBS, Gibco) added in. Chemical reagents used in different cell treatments were purchased from these providers respectively: CHX (cycloheximide), MG132, and BafA1 (bafilomycin A1) from Sigma-Aldrich (USA), human recombinant EGF (epidermal growth factor) from Invitrogen (USA). The cationic lipid reagent (40802ES02) used for plasmid and oligonucleotides transfection was supplied by YEASEN Biotech (China). Reagents shipped in powder were reconstituted following the provider’s instructions.

### Breast cancer samples and staining

The initial 10 cases of breast cancer samples with each 5 of Luminal or TNBC were obtained as FFPE (formaldehyde-fixed and paraffin-embedded) tissues from the Department of Pathology in Sir Run-Run Hospital of Nanjing Medical University, and the following 60 FFPE breast cancer tissues sampled from 30 Luminal and 30 triple-negative breast cancer patients were collected from the Second Affiliated Hospital of Dalian Medical University (Dalian, China). The collection of all breast cancer samples was approved by the ethics committee of each hospital. Antibodies used for IHC (immunohistochemical) staining including anti-HRD1 (ab118483) and anti-Vimentin (ab20346) were purchased from Abcam (USA). After staining, the sections were evaluated and the IHC score of each sample was given by three independent pathologists, calculated as multiplying the percentage of positive cells by the staining intensity, and ultimately categorized into four levels: 0 (−, no staining), 1 (+, weak staining), 2 (++, moderate staining), 3 (+++, deep staining).

### Immunoblotting (IB) and immunoprecipitation (IP)

For protein extraction, cells were lysed in RIPA buffer (Beyotime Biotechnology, China) with 1% Halt™ Protease and Phosphatase Inhibitor Cocktail (Thermo Fisher Scientific, USA) on ice and then centrifuged at 12000 g/4 °C for 15 min to discard pellets. Extracted proteins were denatured by heat (95 °C) with 6x SDS loading buffer (Beyotime Biotechnology) for 10 min before PAGE (polyacrylamide gel electrophoresis). After SDS-PAGE, proteins were transferred onto PVDF membrane and blocked with 5% skim milk solution for 2 h, followed by incubation with primary antibody at 4 °C overnight, finally incubated with secondary antibody at room temperature for 2 h and subjected to enhanced chemiluminescence imaging.

For immunoprecipitation assays, NP-40 lysis buffer with a recipe of 30 ml of 5 M NaCl, 100 ml of 10% NP-40, 50 ml of 1 M Tris (pH 8.0) and 820 ml of H_2_O was used together with 1% Halt™ Protease and Phosphatase Inhibitor Cocktail (Thermo Fisher Scientific) to extract total protein. The whole-cell lysates were centrifuged at 12000 g/4 °C for 15 min to discard pellets. The supernatants, after 10 min of pre-absorption with Pierce™ Protein A/G Agarose (20,421, Thermo Fisher Scientific), were recollected and incubated with specific primary antibody on a rotator overnight at 4 °C. Next, the Protein A/G agarose beads were re-added in to absorb the antigen-antibody complex at 4 °C for 6 h, then recollected and denatured by heat (95 °C) with 2x SDS loading buffer for 10 min. The supernatants from denaturation were finally subjected to immunoblotting.

Primary antibodies used in IB and IP consist of anti-HRD1 (13473–1-AP, Proteintech Group), anti-Vimentin (60330–1-lg, Proteintech Group), HRP-conjugated anti-β-actin (HRP-60008, Proteintech Group), anti-Myc (16286–1-AP, Proteintech Group), anti-FLAG (20543–1-AP, Proteintech Group), anti-HA (51064–2-AP, Proteintech Group), anti-Fibronectin (F3648, Sigma Aldrich), anti-N-Cadherin (BS72312, Bioworld Technology), anti-E-Cadherin (BS72286, Bioworld Technology), anti-TWIST1 (46,702, Cell Signaling Technology), and anti-Snail (3879, Cell Signaling Technology). Additionally, rabbit and mouse IgG isotypes (3900, 5415, Cell Signaling Technology) were used as negative control for immunoprecipitation. Secondary antibodies used in IB include HRP-conjugated anti-mouse/rabbit IgG (SA00001–1/2, Proteintech Group). In particular, HRP-conjugated light chain specific anti-rabbit IgG (SA00001-7 L, Proteintech Group) was used to avoid heavy chain interference.

### Cell immunofluorescence (IF) staining

Having been treated with MG132 (10 μM) for 6 h, HRD1-overexpressing MDA-MB-231 cells seeded in 35 mm glass-bottom dish were rinsed using phosphate-buffered saline (PBS) and then fixed overnight with 4% formaldehyde solution. After fixation, 0.2% Triton X-100 was used for permeabilization of the cell membrane. Fifteen min later, cells were blocked by 1% bovine serum albumin (BSA, Beyotime Biotechnology) at room temperature for 2 h and then primary antibodies anti-HRD1 (13473–1-AP, Proteintech Group) and anti-Vimentin (60330–1-lg, Proteintech Group) were added for overnight incubation at 4 °C. The next day cells were rinsed three times and incubated with fluorophore-tagged secondary antibodies (A32731 and A32727, Invitrogen) at 37 °C for 1 h, successively. Before microscopic imaging, the cell nuclei were stained by DAPI (4′,6-diamidino-2-phenylindole, D9542, Sigma Aldrich) for 5 min, then observed using a ZEISS LSM 900 Confocal Laser Scanning Microscope (ZEISS Microscopy, Germany). The captured images were processed using ZEISS ZEN lite software.

### Reverse transcription PCR (RT-PCR) and RT-qPCR

Total RNA extraction from cells and FFPE tissues was performed using TRIzol™ Plus RNA Purification Kit (12,183,555, Invitrogen) and RNAprep™ pure FFPE Kit (DP439, TIANGEN Biotech, China) respectively according to the manufacturer’s instructions. For circular RNA identification, Linear RNAs were removed by RNase R (RNR07250, Lucigen, USA) after extraction. Isolated and purified RNAs were reverse-transcribed to cDNAs with ReverTra Ace™ qPCR Kit (FSQ-101, TOYOBO Biotech, Japan). Different premixed systems were used for conventional RT-PCR (Green Taq Mix (P131), Vazyme Biotech, China) or Real-Time quantitative RT-PCR (FastStart Universal SYBR Green Master (4,913,850,001, Roche Molecular Systems, Switzerland)). Products of RT-PCR were visualized by electrophoresis on 1.5% agarose gel with Gel-Red (D0139, Beyotime Biotechnology) staining using ChemiDoc™ Imaging System (Bio-Rad, USA). RT-qPCR was carried out using StepOnePlus™ Real-Time PCR System (Applied Biosystems, USA).

### Mass spectrometry

MDA-MB-231 cells stably overexpressing HRD1 were treated with MG132 (10 μM) for 6 h and then lysed in NP-40 lysis buffer containing protease and phosphatase inhibitors. IP was implemented overnight at 4 °C with anti-HRD1 (13473–1-AP, Proteintech Group) or rabbit normal IgG (3900, Cell Signaling Technology). Precipitated proteins were separated through SDS-PAGE, followed by Coomassie blue staining and subsequently sent to the Analysis & Test Center of Nanjing Medical University for liquid chromatography-mass spectrometry (LC-MS) assay. The quantitative proteomics analysis was performed using the MaxQuant software (version 1.6.5.0) and protein intensities were calculated as iBAQ (intensity-based absolute quantification) value.

### Plasmids, lentiviruses, and oligonucleotides

Full-length human SYVN1 and VIM coding sequences were PCR-amplified from MCF-7 cDNA using specific primers. Purified PCR products were subcloned into pcDNA3.1-Myc (V80020, Invitrogen) or pCMV-Flag (D2632, Beyotime Biotechnology) vector, expressing Myc-HRD1 or Flag-Vimentin after cell transfection. Other recombinant plasmids including pcDNA3.1(+), pcDNA3.1-Myc-HRD1(C291S) and pCMV-HA-Ub were kindly provided by the Department of Biochemistry and Molecular Biology of Nanjing Medical University. Luciferase reporter plasmids containing different RNA-RNA interaction sites and their mutated counterparts were generated by Genechem (Shanghai, China) with their GV272 vector. For stable transfection, the lentiviral vector as negative control and recombinant Lenti-HRD1, Lenti-Vimentin, Lenti-circNR3C2 were provided by Genechem as well. MiR-513a-3p mimics and inhibitor were synthesized by GenePharma (Shanghai, China). Sequences of oligonucleotides and primers for PCR amplification were listed in *supplementary materials*.

### CHX chase assay

MDA-MB-231 cells grouped by plasmids transfection (pcDNA3.1(+)/pcDNA3.1-Myc-HRD1/pcDNA3.1-Myc-HRD1(C291S)) were seeded in 6-well plates at a density of 1 × 10^5^ cells per well and cultured overnight. Then, cells were treated with 50 μg/ml of cycloheximide (CHX) and harvested after 0/2/4/6/8/10 h of CHX treatment. The total protein was extracted and subjected to SDS-PAGE to detect the protein level of Vimentin at different time points, indicating Vimentin stability in MDA-MB-231 cells overexpressing wild-type HRD1 or C291S mutant.

### Cell proliferation assay

Prior to proliferation assay, MDA-MB-231 and BT549 cells with different treatments were seeded in 96-well plates at a density of 1000 cells per well, and cultured overnight. Then, Cell Counting Kit-8 (CCK-8, C0037, Beyotime Biotechnology) was used to determine the proliferation rate of each group of cells for every 12 h. The optical density at 450 nm (OD450), in linear correlation with the number of living cells, was measured by Varioskan™ LUX microplate reader (Thermo Fisher Scientific).

### Colony formation assay

Grouped MDA-MB-231 cells were seeded in 6-well plates at a density of 500 cells per well. After 2 or 3 weeks of normal culture, cells were fixed with 4% formaldehyde solution for at least 10 min, then stained with crystal violet staining solution (C0121, Beyotime Biotechnology). Digital photos captured were analyzed via ImageJ (version 1.8.0) to calculate the number of colonies.

### Wound healing assay

MDA-MB-231 and MCF-7 cells were seeded in 6-well plates with 1 × 10^5^ cells per well. Until cell confluency reached about 70% the Eppendorf 50-1000 μl pipette tips were applied to create wound by scratching cells in a straight line. The 0 h images were taken immediately after wound creation via an inverted microscope imaging system (IX-51, Olympus, Japan), and cells were cultured in medium with 1% FBS until 24 h images were taken in the same way. The relative migration rate was calculated as a ratio of 0 h to 24 h scratch width compared to control.

### Cell invasion assay

The Millicell™ hanging cell culture inserts (MCMP24, Millipore, USA) were coated with Matrigel™ Matrix (354,234, Corning, USA) as described in the manufacturer’s protocol. After 24 h of serum-starvation, MDA-MB-231 or MCF-7 cells (5 × 10^4^) were plated in the pre-coated insert in 200 μl serum-free medium, and incubated in a 24-well plate containing 0.75 ml medium with 10% FBS per well for 24 h. With non-invasive cells on the upper surface of the membrane removed with a cotton swab, invasive cells that migrated through the membrane and adhered to the lower surface were fixed with methanol and stained with crystal violet staining solution (Beyotime Biotechnology). The number of invasive cells per field was quantified using an inverted microscope imaging system (IX-51, Olympus).

### Flow cytometry

Grouped MCF-7 cells were detached and dissociated with 0.25% trypsin (Beyotime Biotechnology), centrifuged at 300 x g for 5 min. Cell pellets were resuspended in dedicated staining buffer (420,201, Biolegend, USA) and incubated with FITC anti-human CD44 (338,803, Biolegend) and PE anti-human CD133 (372,803, Biolegend) according to the manufacturer’s staining protocol. Flow cytometry analysis was performed after cell staining using FC500 Flow Cytometer (Beckman Coulter, USA).

### In vitro ubiquitination assay

Myc-HRD1/Myc-HRD1(C291S), Flag-Vimentin and HA-Ub were transfected into different groups of MDA-MB-231 or HEK293 cells treated with 10 μM MG132 for 6 h in advance. Forty-eight h after transfection, protein extraction was performed with NP-40 lysis buffer and the whole-cell lysates were incubated in rotation with anti-Flag (20543–1-AP, Proteintech Group) or rabbit IgG isotype (3900, Cell Signaling Technology) at 4 °C overnight. Precipitation of the antibody-bound proteins by Pierce™ Protein A/G Agarose (20,421, Thermo Fisher Scientific) was accomplished afterward. The precipitates were washed with washing buffer (50 mM Tris pH 8.5, 1 mM EGTA, 75 mM KCl) and boiled with 2x SDS loading buffer for 5 min to elute proteins projected to SDS-PAGE and immunoblotting analysis.

### Generation of stable cell lines

Lenti-HRD1, Lenti-Vimentin and Lenti-circNR3C2 were added separately into MDA-MB-231 cells seeded in 6-well plates at a density of 1 × 10^5^ cells per well, according to the user manual of lentivirus provided by Genechem. Twelve h after infection, cells were re-plated in 24-well plates with complete medium, and puromycin (2 μg/ml) or G-418 (0.5 mg/ml) was used for selection of stable cells. When the control cells without lentivirus infection were all killed by antibiotics, puromycin or G-418 was reduced from selection concentration to sustaining concentration (1/3 of the former) and the infection efficiency was examined by observing the green fluorescence intensity. Once fully infected, stable cells were cryopreserved or used for other experiments and assays.

### Fluorescence in situ hybridization

To investigate the intracellular distribution of circNR3C2 in breast cancer, a fluorescence in situ hybridization kit (F03301) from GenePharma was used following the manufacturer’s instructions. In brief, MDA-MB-231 and MCF-7 cells were seeded in 35 mm glass-bottom dishes at a density of 1 × 10^5^ cells per dish and cultured overnight, then fixed with 4% formaldehyde solution, washed with SSC (saline sodium citrate) buffer and permeabilized with 0.1% Triton X-100 solution, finally incubated with fluorophore (6FAM)-labeled circNR3C2 probes for hybridization at 37 °C overnight. The 6FAM-labeled circNR3C2 probes were also obtained from GenePharma. After hybridization, the images were taken by ZEISS LSM 900 Confocal Laser Scanning Microscope (ZEISS Microscopy). Sequences of circNR3C2 and positive/negative control probes were shown in *Supplementary materials*.

### Biotinylated RNA pull-down assay

The biotinylated DNA probes, synthesized by RioBio (Guangzhou, China) for circNR3C2 (sequence: GTCTCCATCGCTTGATACAT) and negative control (sequence: TCTGTTCGTTAGGTCCCTGG), were dissolved in DNase/RNase-free water to 100 μM. The probes were incubated with lysates of MDA-MB-231 and BT549 cells overexpressing circNR3C2 at 37 °C for 4 h, after which streptavidin-coated magnetic beads (Dynabeads™ MyOne™ Streptavidin C1, 65,001, Invitrogen) were added in for an extra incubation at 37 °C for 30 min. C1 beads were rinsed with wash buffer and then the pull-down complex was eluted, from which RNAs were extracted using TRIzol for RT-qPCR analysis.

### Luciferase reporter assay

HEK293 cells were co-transfected with the GV272 luciferase reporter vectors harbouring wild-type or mutated binding sites of miR-513a-3p on HRD1 3’UTR or circNR3C2 and pRL Renilla Luciferase Control Reporter Vectors (E2241, Promega, USA) as internal control. At the same time, cells were transfected with miR-513a-3p mimics/inhibitor or negative control oligonucleotides and cultured continuously for 48 h. Then cells were lysed for luciferase reporter assay using a Dual-Luciferase Reporter Assay Kit (E1910, Promega). The relative firefly-luciferase activity was measured by testing the bioluminescence with Varioskan™ LUX microplate reader (Thermo Fisher Scientific) and normalized by the renilla-luciferase activity.

### In vivo tumor formation

6-week old female BALB/C nude mice were purchased from CAVENS LAB ANIMAL (China). For in vivo tumor formation, MDA-MB-231 cells, either stably overexpressing circNR3C2 or circNR3C2/Vimentin, were resuspended in 1:1 PBS/Matrigel (356,237, Corning) solution and injected subcutaneously in the flank of nude mice at a density of 5 × 10^6^ cells in 0.1 ml per mouse. Mice were kept in a specific-pathogen-free (SPF) environment and tumor size was measured weekly since 2 w after injection. Six weeks after injection, euthanasia was administered and tumors were isolated with the volume calculated according to the formula *Volume* = (*long diameter* x *short diameter*^2^)/2. All procedures were approved by the Institutional Animal Care and Use Committee of Nanjing Medical University.

### In vivo metastasis model

6-week old female BALB/C nude mice were purchased from CAVENS LAB ANIMAL (China). For in vivo metastasis model, MDA-MB-231 cells, either stably overexpressing circNR3C2 or circNR3C2/Vimentin, were injected into nude mice through the tail vein at a density of 2 × 10^6^ cells in 0.15 ml per mouse. Mice were kept in a specific-pathogen-free (SPF) environment and the body weight was weekly examined. Five weeks after intravenous injection, euthanasia was administered and the lungs were dissected for counting the number of metastatic nodules and photo taking. Lungs with tumor tissues were fixed in 4% formaldehyde solution and embedded in paraffin wax blocks for sectioning. Tissue sections were mounted on slides and stained with hematoxylin and eosin (H&E), histologically evaluated with an Eclipse 80i digital microscope (Nikon, Japan). All procedures were approved by the Institutional Animal Care and Use Committee of Nanjing Medical University.

### Differential expression analysis

Sixty nine thousand eight hundred-fifteen distinct circular RNAs were identified in GSE113230 and those with average RPM < 0.1 and total back-spliced reads < 2 were discarded. Differential expression analysis was performed on the remaining circular RNAs using DEGseq R package. In summary, with a criterion of foldchange ≥2 and *P* < 0.05, 5033 circular RNAs were identified as differentially expressed between TNBC and adjacent normal tissues, among which 3726 circRNAs were significantly downregulated and 1307 circRNAs were significantly upregulated in TNBC tissues.

### MiRNA prediction

MiRNAs that potentially bind to the 3′ untranslated region (3’UTR) of HRD1 mRNA were predicted using 5 different algorithms including miRMap (https://mirmap.ezlab.org/), miRanda (http://www.microrna.org/microrna/home.do), microT-CDS (http://diana.imis.athena-innovation.gr/DianaTools/index.php?r=microT_CDS/index), miRDB (http://www.mirdb.org/) and Targetscan (http://www.targetscan.org/vert_72/). MiRNAs potentially sponged by circNR3C2 were predicted via Circinteractome (https://circinteractome.nia.nih.gov/). The overlap of these two parts of miRNAs was chosen as miRNA candidates sponged by circNR3C2 and silencing HRD1 mRNA in a high probability.

### Enrichment analysis

Enrichment analysis of miRNA candidates was based on their target genes. DAVID Bioinformatics Resources 6.8 (https://david.ncifcrf.gov/) was used to figure out whether the target genes were enriched in molecular functions, biological processes and pathways associated with our study. MirPath v3 (http://snf-515788.vm.okeanos.grnet.gr/) was used for target genes prediction and GO (Gene Ontology)/KEGG (Kyoto Encyclopedia of Genes and Genomes) pathway enrichment analysis of miRNA candidates.

### Statistical analysis

All experiments were repeated at least three times in this study. GraphPad Prism (version 8.2.0) and Microsoft Excel 2016 were used for statistical analysis. One-way analysis of variance (ANOVA) was used for comparison of different breast cancer subtypes in TCGA-BRCA project. The Kaplan-Meier estimator and log-rank test were used to analyze the relapse-free survival. Pearson or Spearman’s correlation coefficient was applied to measure linear correlation between two groups of variables. Fisher’s exact test was used for assessment of clinicopathologic significance between two groups of categorical samples. Unpaired two-tailed Student’s t-test was used for all other hypothesis testing. Quantitative data are expressed as mean value ± standard error and *P* < 0.05 was considered statistically significant.

## Results

### HRD1 is significantly underexpressed in TNBC

**Fig. 1:**
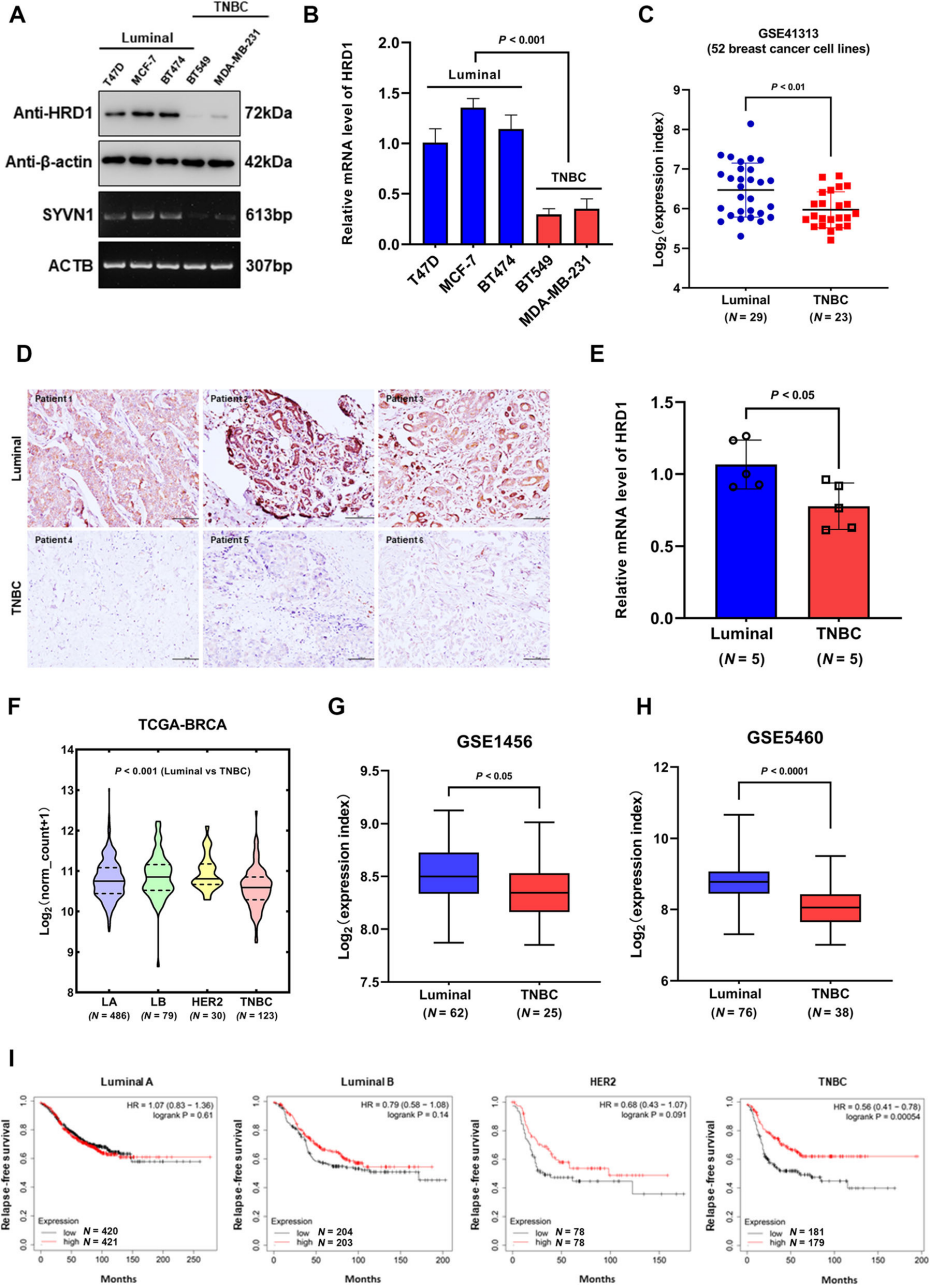
Expression profile of HRD1 in different subtypes of breast cancer. **a** Expression of HRD1 at the protein and mRNA level in luminal (T47D, MCF-7, BT474) and basal-like (MDA-MB-231, BT549) breast cancer cell lines, examined by immunoblotting and RT-PCR with β-actin as a loading control. **b** RT-qPCR results of HRD1 expression in five kinds of breast cancer cells. β-actin was used as an internal control. **c** Scatter plots of HRD1 expression in 52 breast cancer cell lines from public microarray dataset (GSE41313). Luminal *N* = 29, TNBC *N* = 23. **d** Representative immunohistochemical staining of HRD1 protein expression in luminal breast cancer and TNBC tissues. Scale bar = 50 μm. **e** RT-qPCR results of HRD1 mRNA level in FFPE luminal (*N* = 5) and TNBC (*N* = 5) tissues. β-actin was used as an internal control. **f** Violin plots demonstrating the differential expression of HRD1 across the breast cancer subtypes, sampling from The Cancer Genome Atlas (TCGA-BRCA). LA (Luminal A) *N* = 486, LB (Luminal B) *N* = 79, HER2 *N* = 30, TNBC *N* = 123. One-way ANOVA was used for statistical analysis. **g**, **h** Box plots showing HRD1 expression in patients with luminal breast cancer or TNBC from public microarray datasets (GSE1456 and GSE5460). Luminal *N* = 62/76, TNBC *N* = 25/38. **i** Kaplan-Meier analysis showing the influence of HRD1 expression on relapse-free survival across the breast cancer subtypes from multiple public microarray datasets collected and organized by *KM plotter*. Data were represented as means ± S.D. of at least three independent experiments

In our previous study, the lower expression of HRD1 was observed in breast cancer tissues compared to adjacent normal breast tissues [13]. To further investigate the exact expression profile of HRD1 in different breast cancer subtypes, we initially examined the protein and mRNA level in three breast cancer cell lines of luminal subtype (MCF-7, T47D, BT-474) and two basal-like (TNBC) cell lines including MDA-MB-231 and BT549 [18]. Immunoblotting and RT-PCR showed that HRD1 is significantly underexpressed in TNBC cell lines in comparison with luminal breast cancer cells whether at protein or mRNA level (Fig. 1a), which was supported by quantitative RT-PCR analysis (Fig. 1b). Furthermore, a public microarray dataset (GSE41313) containing gene expression data of 52 kinds of breast cancer cell lines [19] was analyzed with GEO2R, indicating a remarkably lower expression of HRD1 in TNBC cells against luminal cells (Fig. 1c). Then we assessed the protein and mRNA level of HRD1 in 10 cases of FFPE breast cancer tissues classified as 5 luminal and 5 TNBC with immunohistochemical staining and RT-qPCR. In accordance with the expression profile of cell lines, the protein and mRNA of HRD1 presented a fairly lower expression pattern in TNBC tissues rather than those with luminal subtype (Fig. 1d, e). Moreover, these findings were corroborated by the analysis of public RNA-sequencing (TCGA-BRCA) and microarray (GSE1456, GSE5460) [20, 21] datasets (Fig. 1f-1h). To evaluate the associations between HRD1 expression and the clinical outcomes of breast cancer patients with different subtypes, we performed Kaplan-Meier survival estimates using the KM-Plotter tool [22]. Notably, patients with high HRD1 expression exhibited significantly longer relapse-free survival than those with low expression (Supplementary Fig. 1), while this correlation only existed in TNBC when analyzed separately by subgroup (Fig. 1i). Taken together, these results suggest that HRD1 is in close association with invasive and aggressive breast cancer subtype and may serve as a predictive factor for tumor progression.

### HRD1 expression is in negative correlation with Vimentin

**Fig. 2:**
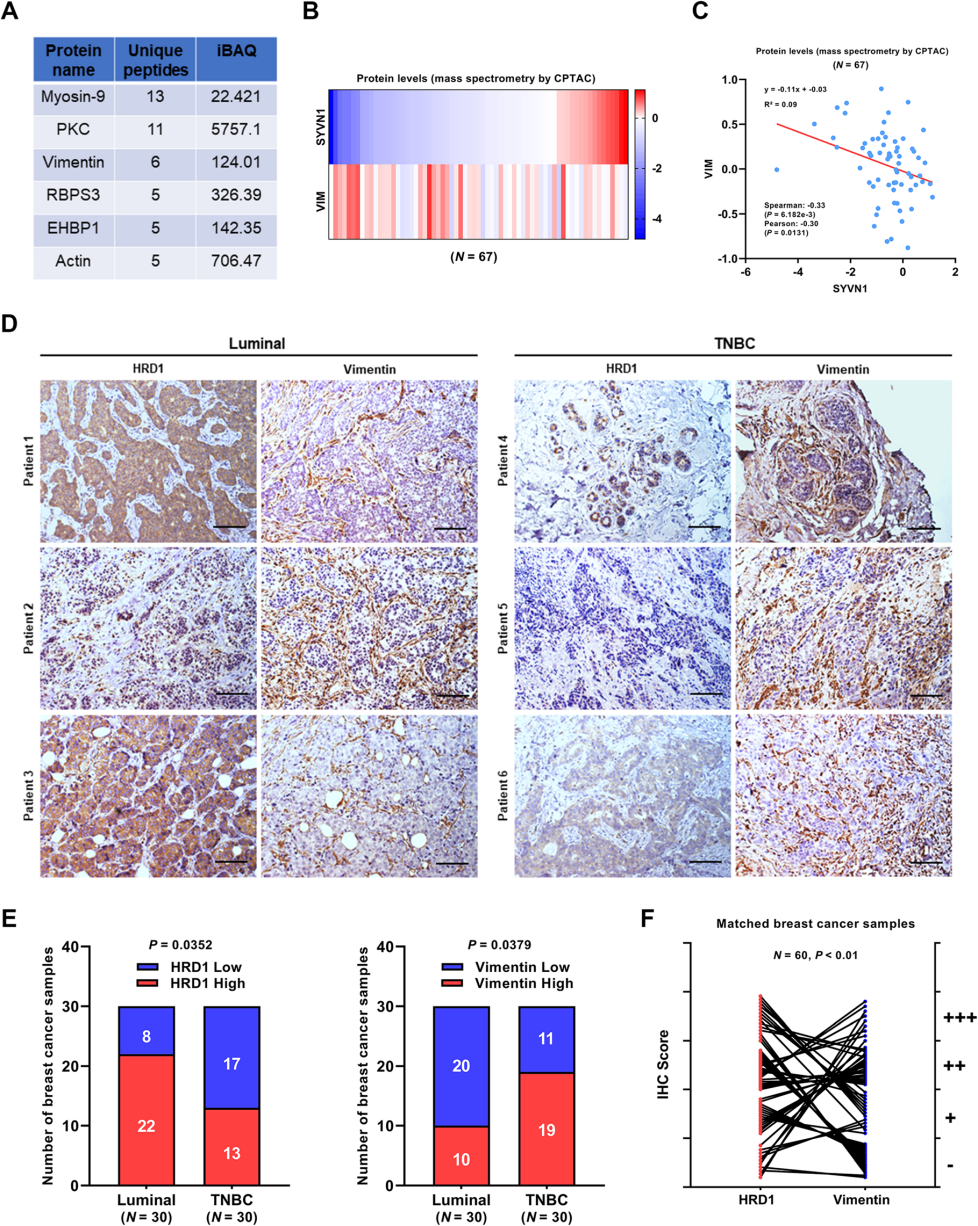
Correlation between HRD1 and Vimentin protein expression in breast cancer. **a** list of the top 6 HRD1-binding protein candidates in MDA-MB-231 cells, analyzed by liquid chromatography-mass spectrometry assay. iBAQ = intensity-based absolute quantification. **b** Heatmap showing the protein expression of HRD1 and Vimentin assessed with mass spectrometry in breast cancer samples (*N* = 67) from the Clinical Proteomic Tumor Analysis Consortium (CPTAC). Color key = protein expression Z-score. **c** Correlation between HRD1 and Vimentin protein expression in breast cancer samples (*N* = 67) from CPTAC, determined by Pearson’s correlation coefficient. **d** Representative immunohistochemical staining of HRD1 and Vimentin protein expression in both luminal subtype and TNBC tissues. Scale bar = 50 μm. **e** Stacked bar charts exhibiting the distribution of breast cancer samples (*N* = 60) with different expression levels of HRD1 and Vimentin evaluated by the IHC score. 0 (−) and 1 (+) were regarded as low expression, while 2 (++) and 3 (+++) were regarded as high expression. Luminal *N* = 30, TNBC *N* = 30. Fisher’s exact test was performed for statistical analysis. **f** Paired line plots showing the IHC score of HRD1 and Vimentin in matched breast cancer tissue sections (*N* = 60). *P* value was calculated as Pearson’s correlation coefficient, showing a reverse correlation of HRD1 and Vimentin

Considering the intrinsic nature of HRD1 as an E3 ubiquitin ligase [23], we implemented a liquid chromatography-mass spectrometry assay in HRD1-overexpressing MDA-MB-231 cells to find out the underlying mechanisms of HRD1 functions and its potential substrates in triple-negative breast cancer. As an important intermediate filament family member whose expression was frequently caught in multiple malignant tumors especially TNBC, Vimentin was identified as a potential binding protein of HRD1 with high probability (Fig. 2a). Overexpressed in several aggressive breast cancer cell lines where HRD1 was verified to be significantly downregulated (Fig. 1a, b), Vimentin plays a pivotal role at the very center of the EMT process through which the increased cellular invasion and migration lead to the metastasis of breast cancer [9, 24]. Thus, to determine whether there is an association between HRD1 and Vimentin at the protein level, we performed proteomic analysis on mass spectrometry data from the Clinical Proteomic Tumor Analysis Consortium (Fig. 2b) and found that HRD1 expression is in a prominently negative correlation with Vimentin in breast cancer samples of the CTPAC project (Fig. 2c). Additionally, 60 FFPE breast cancer tissues obtained from the Second Hospital of Dalian Medical University were inspected with immunohistochemical staining to confirm the relevance between HRD1 and Vimentin expression. No matter luminal or TNBC they were, sections with high expression of HRD1 or areas where positive HRD1 staining distributed tended to have a relatively lower staining of Vimentin and vice versa (Fig. 2d, e). Together with the IHC score for HRD1 and Vimentin in matched breast cancer samples (Fig. 2f), these results suggest a significant inverse relationship between HRD1 and Vimentin expression in patients with breast cancer.

### HRD1 mediates Vimentin degradation through the ubiquitin-proteasome system

**Fig. 3:**
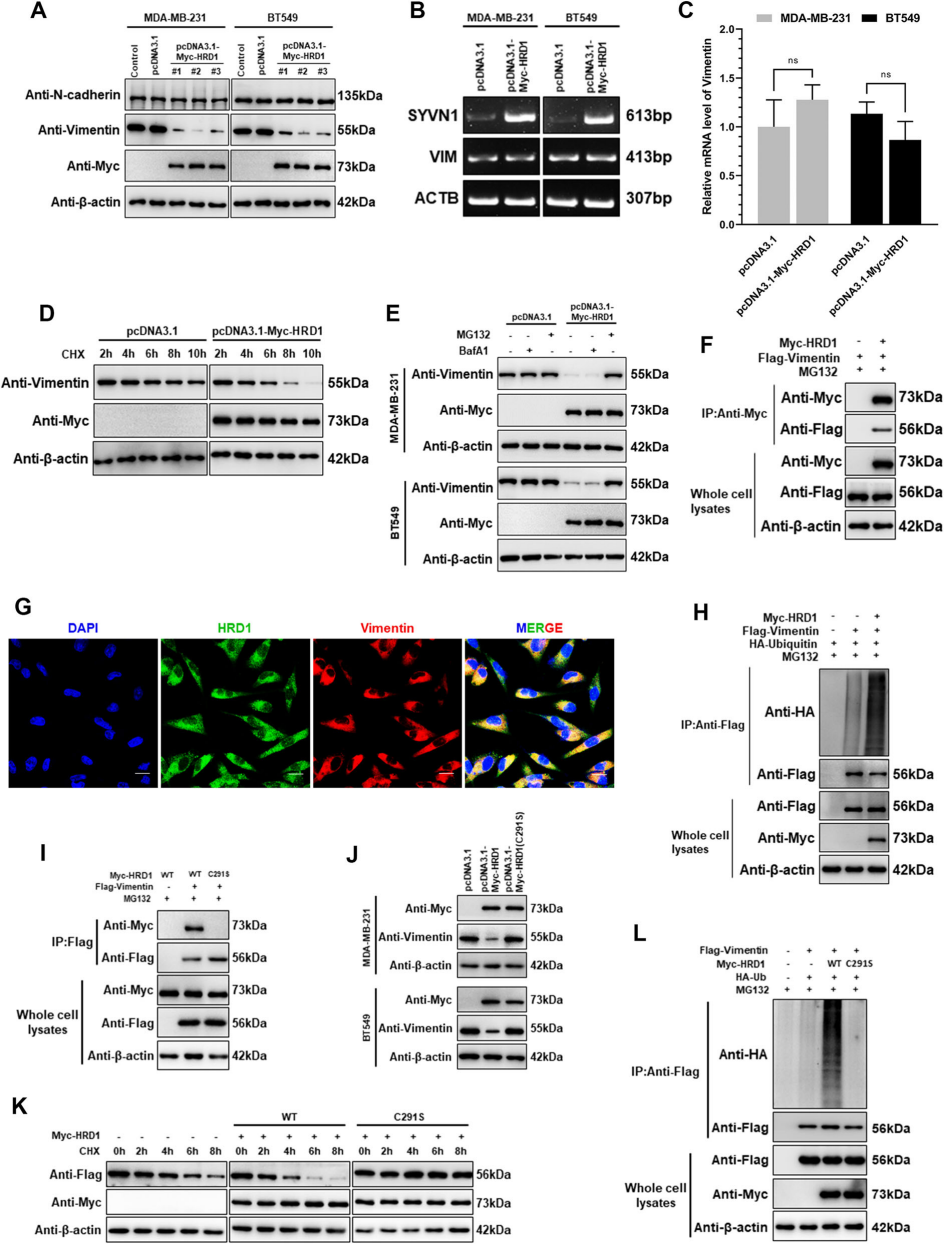
HRD1 targets Vimentin for polyubiquitination-mediated degradation. **a** Immunoblotting showing the expression of N-Cadherin and Vimentin in control cells and HRD1-overexpressing MDA-MB-231 and BT549 cell clones (#1-#3) with β-actin as a loading control. **b** Agarose gel electrophoresis of RT-PCR products showing the mRNA expression of HRD1 and Vimentin in control and HRD1-overexpressing MDA-MB-231 and BT549 cells. Transcript of β-actin was used as a loading control. **c** Vimentin mRNA level in MDA-MB-231 and BT549 cells before and after HRD1 overexpression, examined with real-time quantitative RT-PCR. β-actin was used as an internal control. **d** Stability tests of Vimentin in control and HRD1-overexpressing MDA-MB-231 cells treated with 50 μg/ml of cycloheximide (CHX) for the indicated times. β-actin was used as a loading control. **e I**mmunoblotting showing the expression of Vimentin in control and HRD1-overexpression MDA-MB-231 and BT549 cells pretreated with MG132 (10 μM) or Bafilomycin A1 (BafA1, 100 nM) for 6 h. β-actin was used as a loading control. **f** Endogenous interaction between HRD1 and Vimentin detected by in vitro co-immunoprecipitation assay in HEK293 cells cotransfected with Myc-HRD1 and Flag-Vimentin plasmids upon MG132 (10 μM) treatment. Whole-cell lysates were immunoprecipitated with anti-Myc antibody, then immunoblotted and hybridized with the indicated antibodies. **g** Immunofluorescence staining showing a co-localization of HRD1 and Vimentin in the cytoplasm of MDA-MB-231 cells transfected with Myc-HRD1 plasmids upon MG132 (10 μM) treatment. Fluorophore-conjugated secondary antibodies were used as green indicating HRD1 and red indicating Vimentin. DAPI was used for staining the nucleus. Images were taken with a laser scanning confocal microscope. Scale bar = 20 μm. **h** Ubiquitination of Vimentin in MDA-MB-231 cells transfected with HA-ubiquitin, Myc-HRD1 or Flag-Vimentin plasmids upon MG132 (10 μM) treatment. Whole-cell lysates were immunoprecipitated with anti-Flag antibody, then immunoblotted and hybridized with the indicated antibodies. **i** Whole-cell lysates of HEK293 cells cotransfected with Flag-Vimentin and Myc-HRD1 or Myc-HRD1 (C291S) plasmids upon MG132 (10 μM) treatment were immunoprecipitated with anti-Flag antibody, showing the interaction between Vimentin and wild-type HRD1 or C291S mutant. **j** Immunoblotting showing the expression of Vimentin in MDA-MB-231 and BT549 cells before and after the overexpression of wild-type HRD1 or C291S mutant. **k** Stability tests of Vimentin in MDA-MB-231 cells overexpressing wild-type HRD1 or C291 mutant, treated with CHX (50 μg/ml) for the indicated times. **l** In vitro ubiquitination assay of Vimentin similar to Fig. 3**h**, with the addition of HRD1 (C291S) overexpression

Based on the results mentioned above, it is reasonable for us to hypothesize that HRD1 could ubiquitinate Vimentin and lead to its degradation through proteasome in breast cancer. To verify this hypothesis, we first assessed the impact of HRD1 on Vimentin stability. HRD1 overexpression in MDA-MB-231 and BT549 cells conspicuously and steadily reduced the protein level of Vimentin without affecting its mRNA expression and other EMT markers, like N-Cadherin (Fig. 3a-3c). Moreover, the CHX chase assay revealed that overexpression of HRD1 caused a faster degradation rate of Vimentin in MDA-MB-231 cells pretreated with protein synthesis inhibitor cycloheximide (Fig. 3d). Besides, the primary and enhanced degradation of Vimentin under HRD1 overexpression could be blocked by proteasome inhibitor MG132 but not by autophagy inhibitor Bafilomycin A1 (BafA1), which means that HRD1-mediated Vimentin degradation is fulfilled through proteasome rather than lysosome (Fig. 3e).

Since Vimentin was identified as one of the major binding proteins of HRD1, we next verified the interaction between HRD1 and Vimentin in HEK293 and MDA-MB-231 cells using co-immunoprecipitation and immunofluorescence staining. Co-IP assay showed that ectopic overexpressed HRD1 and Vimentin bound to each other directly in HEK293 cells (Fig. 3f) while IF staining demonstrated a colocalization in the cytoplasm of MDA-MB-231 cells (Fig. 3g). Given that HRD1 ordinarily acts as an E3 ligase transferring ubiquitin to substrate proteins, we performed in vitro ubiquitination assay to check out whether HRD1 mediates the ubiquitination of Vimentin. As expected, overexpression of HRD1 significantly boosted the polyubiquitination level of Vimentin in MDA-MB-231 cells (Fig. 3h).

It’s widely known that E3 ligase activity commonly and mostly relies on the RING finger domain, or more precisely, the cysteine residues that simultaneously binds the ubiquitination enzyme itself and its substrates [25]. According to the protein structure information from UniProt (Q86TM6), HRD1 has a RING-type zinc finger domain containing amino acid residues from the 291th to 330th site. Thus, we generated a HRD1 mutant (C291S) in which the cysteine at the 291th site was substituted with serine to examine whether the RING domain of HRD1 is responsible for binding and ubiquitination of Vimentin. Unsurprisingly, the inactive HRD1 mutant (C291S) was unable to bind Vimentin and to decrease its expression level (Fig. 3i, j), therefore could not mediate the ubiquitination and degradation of Vimentin as well (Fig. 3k, l).

### HRD1 overexpression attenuates tumor progression and EMT progress via Vimentin degradation

**Fig. 4:**
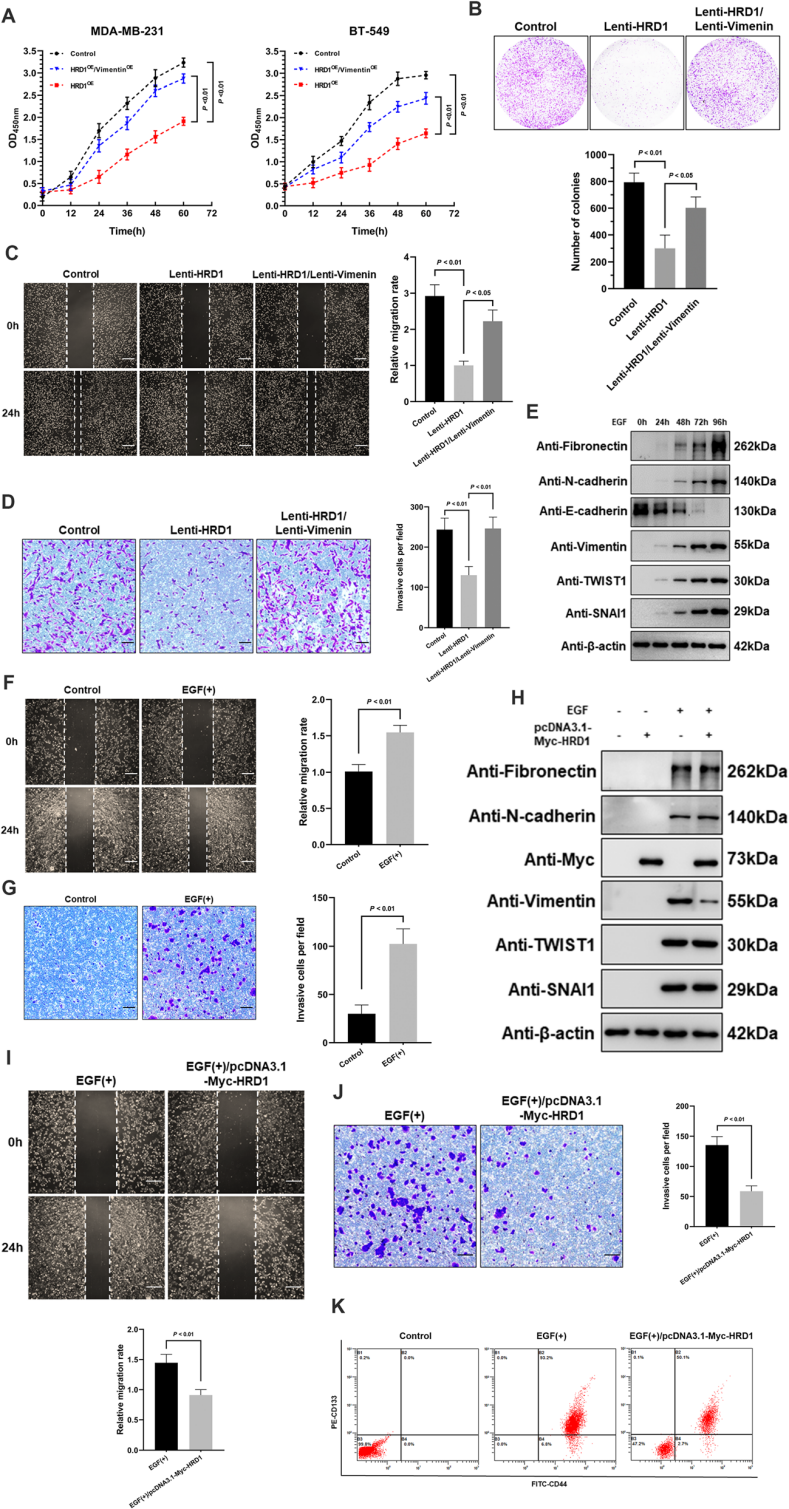
HRD1-mediated Vimentin degradation suppresses TNBC progression and inhibits EMT of breast cancer. **a** CCK-8 assay showing the optical density at 450 nm of MDA-MB-231 and BT549 cells stably overexpressing HRD1/Vimentin or singly HRD1, suggesting the number of living cells at the indicated timepoints. **b** Colonies formed by MDA-MB-231 cells stably overexpressing HRD1/Vimentin or singly HRD1 were stained with crystal violet solution, 15 days after seeding. **c** Cell migration indicated by the scratch width of MDA-MB-231 cells stably overexpressing HRD1/Vimentin or singly HRD1, 0 and 24 h after wound generation. Scale bar = 200 μm. **d** MDA-MB-231 cells stably overexpressing HRD1/Vimentin or singly HRD1, moving through matrigel in 12 h and adhering to the lower surface of the bottom membrane, were stained with crystal violet solution in transwell invasion assay. Scale bar = 100 μm. **e** Immunoblotting showing the expression of epithelial marker (E-Cadherin) and mesenchymal markers (Fibronectin, N-Cadherin, Vimentin, TWIST1 and SNAI1) in MCF-7 cells treated with 30 ng/ml of EGF for indicated times. β-actin was used as a loading control. **f**, **g** Wound healing and transwell invasion assay showing the migratory and invasive capability of MCF-7 cells treated with EGF for 96 h compared to control cells. Scale bar = 200/100 μm. **h** Immunoblotting showing the expression of EMT markers in control and HRD1-overexpressing MCF-7 cells upon EGF treatment. β-actin was used as a loading control. **i**, **j** Wound healing and transwell invasion assay showing the migratory and invasive capability of control and HRD1-overexpressing MCF-7 cells upon EGF treatment. **k** Flow cytometry showing the proportion of cancer stem cells (CD44+ and CD133+) in MCF-7 cells with or without HRD1 overexpression upon EGF treatment. The width of scratch, number of colonies and invasive cells per field were measured using ImageJ. Data were represented as means ± S.D. of at least three independent experiments

Vimentin is abundantly expressed in several aggressive cancer cell lines, and broadly reported to play a critical role in acquisition of the migratory and invasive tumor cell phenotype during the epithelial-mesenchymal transition [9]. Hence, we continued to explore the tumor-suppressive mechanisms of HRD1 targeting Vimentin degradation. Interestingly, the CCK-8 and colony formation assays showed a substantial suppression of cell proliferation and reproductive capability under ectopic HRD1 expression in TNBC cells, which was rescued by overexpression of Vimentin (Fig. 4a, b). Besides, the migratory and invasive capacities were also assessed to be notably weakened in HRD1-overexpressing TNBC cells due to the loss of Vimentin stability, as implied by wound healing and transwell invasion assays (Fig. 4c, d).

In parallel with these results, we build an in vitro EMT model utilizing the luminal breast cancer cell line MCF-7 to further testify the effect of HRD1-mediated Vimentin degradation. Stimulated with recombinant human epidermal growth factor (EGF) as described previously [26], MCF-7 cells underwent an obvious time-lapse transition from epithelial to mesenchymal phenotype, judged from various protein markers including Vimentin (Fig. 4e), exhibiting a stronger tendency to migrate and invade (Fig. 4f, g). Consistently, HRD1 overexpression fairly decreased the elevated protein level of Vimentin through EMT induction but had no influence on the other markers (Fig. 4h), thus the original nonaggressive properties of MCF-7 cells were retrieved (Fig. 4i, j). Since EMT is closely associated with the acquisition of stemness in both normal and neoplastic cells [27], we conducted flow cytometry assays to check the specific stemness markers (CD44 and CD133) in EGF-treated MCF-7 cells. In consequence, the stem cell traits acquired during EMT were partially reversed once accompanied by HRD1 overexpression (Fig. 4k), suggesting that HRD1-induced decline of Vimentin stability could prevent the generation of metastatic breast cancer stem cells (BCSCs).

### CircNR3C2 and miR-513a-3p are predicted to regulate HRD1 in breast cancer

**Fig. 5:**
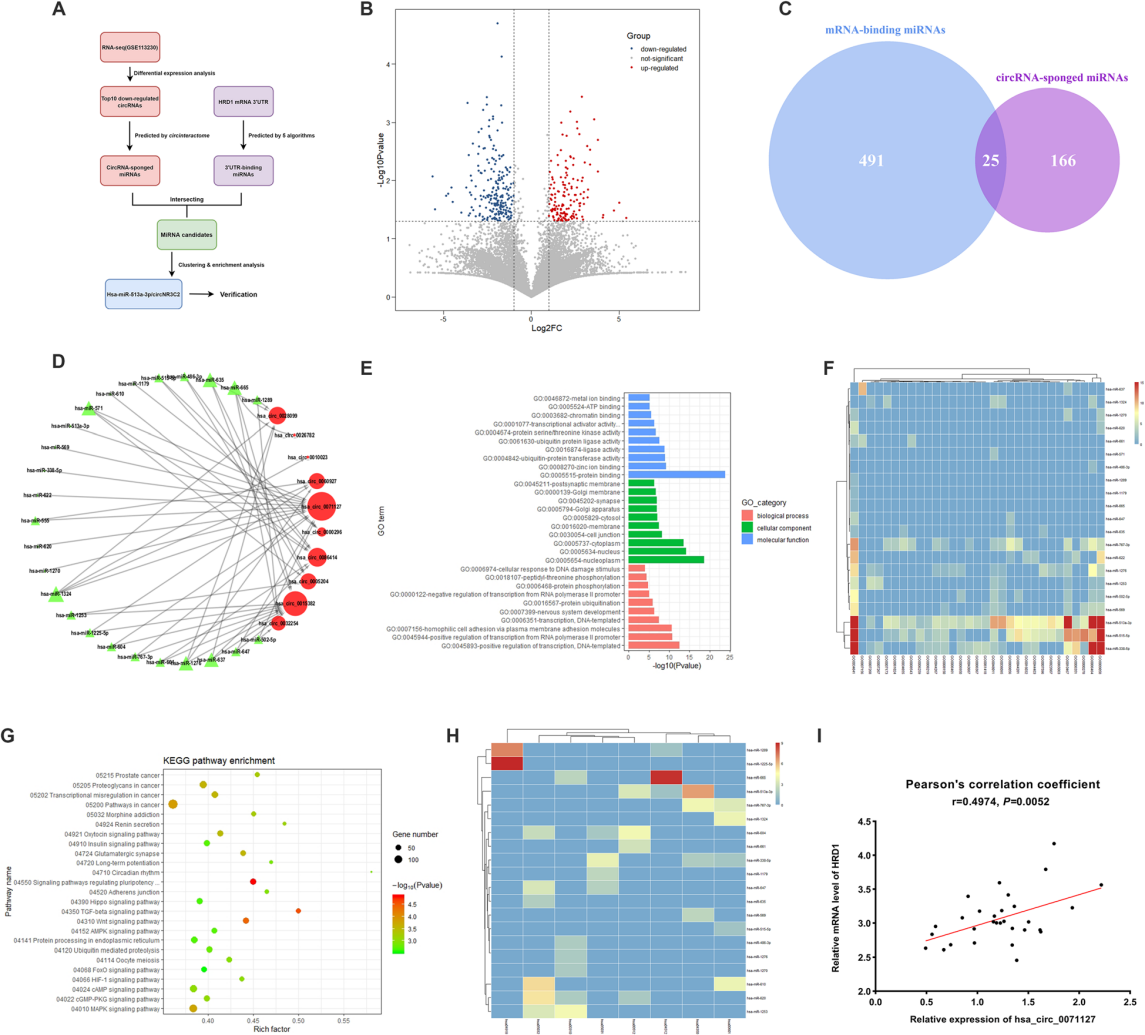
Bioinformatics prediction of circNR3C2 and hsa-miR-513a-3p. **a** Flowchart showing the prediction procedures of circular RNAs and miRNAs possibly regulating HRD1 expression in breast cancer. **b** Volcano plot showing an overview of the differential expression of circular RNAs identified in GSE113230, in 3 TNBC tissues compared to adjacent normal tissues. The upregulated and downregulated circRNAs in red and blue respectively were characterized as fold change (FC) ≥ 2 or ≤ 0.5 with adjusted *P* value < 0.05. **c** Venn diagram showing the overlap between the predicted mRNA-binding miRNAs and circRNA-sponged miRNAs, namely the miRNA candidates predicted to bind to HRD1 mRNA and sponged by the top 10 downregulated circRNAs in TNBC. **d** Interaction network of the circRNA and miRNA candidates. Size of the representative shapes (circle and triangle) depended on the node degree analyzed by Cytoscape software. **e** Gene Ontology (GO) enrichment analysis showing the biological processes and molecular functions (especially GO:0061630 and GO:0016567) in which genes targeted by the miRNA candidates were significantly enriched. **f** Heatmap and hierarchical clustering analysis showing the enrichment of miRNA candidates in the indicated biological processes with corresponding statistical significance. GO:0043687, post-translational protein modification. GO:0006464, cellular protein modification process. GO:0007173, epidermal growth factor signaling pathway. GO:0044267, cellular protein metabolic process. The color key reflects the *P* value. **g** KEGG pathway enrichment analysis showing the biological pathways in which genes targeted by the miRNA candidates were significantly enriched. **h** Heatmap and hierarchical clustering analysis showing the enrichment of miRNA candidates in the indicated biological pathway with corresponding statistical significance. Hsa04350, TGF-beta signaling pathway. The color key reflects the *P* value. **i** Correlation between the expression of circNR3C2 and HRD1 in 30 TNBC tissues

Together with our previously published research [13], We had further confirmed that HRD1 plays a suppressive role in breast cancer in the current study. Here we asked the question, what causes the loss of expression and function of HRD1 during the progression of breast cancer. The regulatory functions of circular RNAs in cancer have been gradually investigated. Particularly, circular RNAs act as an miRNA sponge to absorb specific miRNAs and subsequently diminish their silencing effect on target genes [28]. Combining the existing data in this study, we assumed that the disturbance of circRNA-miRNA axis during tumorigenesis perhaps resulted in the downregulation of HRD1 in TNBC. A set of analysis procedures was used in order to predict the circRNA and miRNA that may regulate HRD1 expression (Fig. 5a). At the beginning, based on the expression matrix of public dataset GSE113230 that contains high-throughput sequencing results of circRNAs in paired TNBC and adjacent normal breast tissues [17], we performed a differential expression analysis with an R package named DEGseq [29]. In total, 5033 differentially expressed circRNAs were identified with a criterion of foldchange ≥2 or ≤ 0.5 and adjusted *p*-value < 0.05, including 1307 upregulated and 3726 downregulated circRNAs in TNBC tissues (Fig. 5b). Next, the top 10 of significantly underexpressed circRNAs were chosen for prediction of sponged miRNAs with *circinteractome* [30], while miRNAs potentially interacting with the 3’UTR of HRD1 mRNA were predicted with five different algorithms mentioned in *materials and methods*. Consequently, 25 miRNAs overlapping between the two groups of predicted results were characterized as miRNA candidates for the following analyses (Fig. 5c).

By far we had built a circRNA-miRNA interaction network (Fig. 5d). To determine the potential circRNA-miRNA combination that specifically regulates HRD1 expression in TNBC, we performed Gene Ontology (GO) and KEGG pathway enrichment analyses on miRNA candidates and their target genes using *DAVID* [31] and *mirPath v3* [32]. Notably, significant enrichment in molecular function (*GO: 0061630, ubiquitin protein ligase activity*), biological process (*GO: 0016567, protein ubiquitination*) and pathways (*hsa04350/04310, TGF-beta/Wnt signaling pathway; hsa04120, ubiquitin-mediated proteolysis*) was observed (Fig. 5e, g). Among them, miR-513a-3p was found enriched with the highest significance in these biological processes and pathways related with HRD1 activity and EMT process (Fig. 5f, h).

Since circNR3C2 (hsa_circ_0071127) was predicted interacting with miR-513a-3p, we testified if there was a correlation between the expression of circNR3C2 and HRD1 in 30 FFPE TNBC tissues. RT-qPCR showed that circNR3C2 had a significant correlation with HRD1 mRNA level (Fig. 5i), which attracted our attention on the circNR3C2/miR-513a-3p/HRD1 axis and its possible impact on tumor progression.

### CircNR3C2 is significantly underexpressed in TNBC

**Fig. 6:**
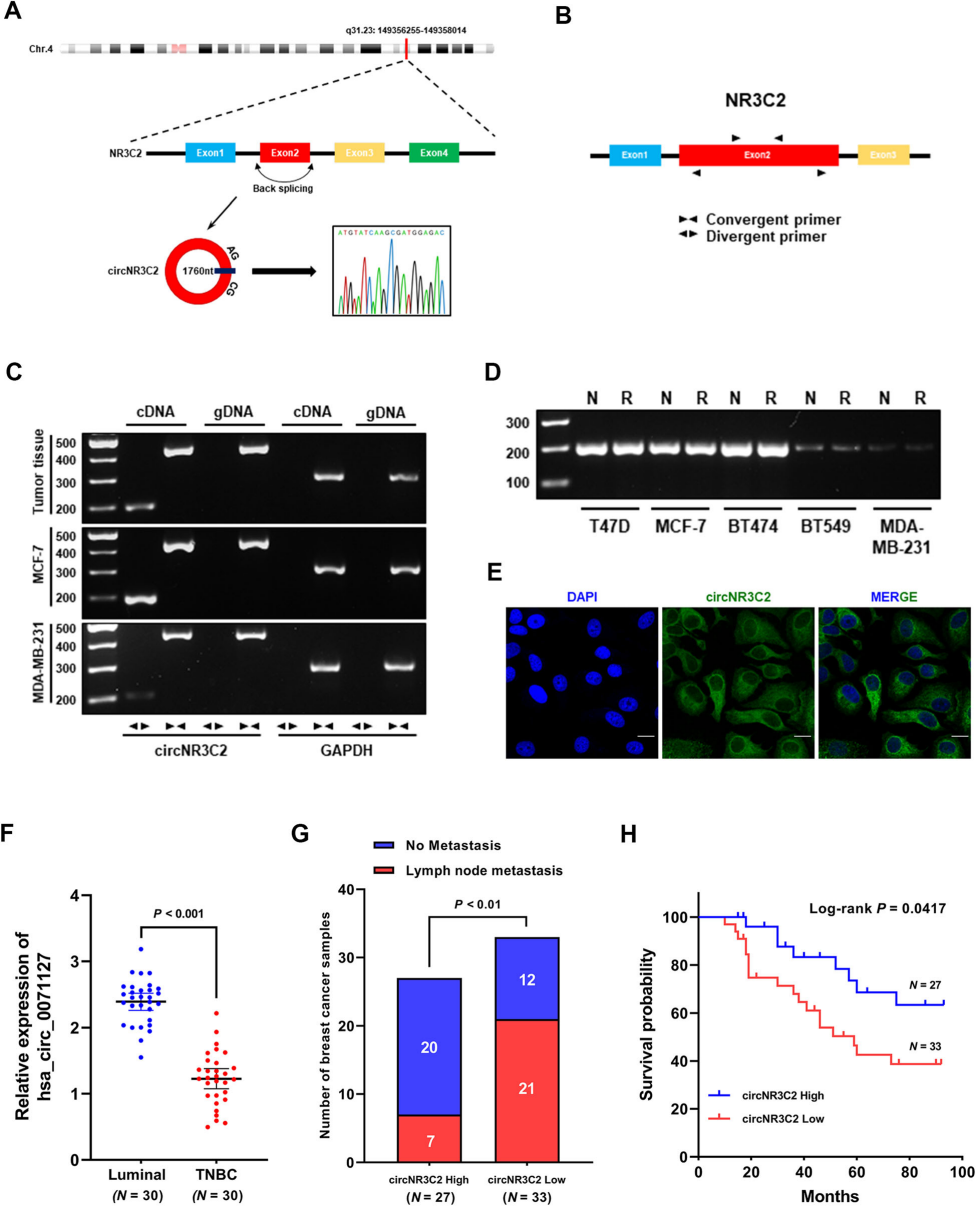
Verification of circNR3C2 in breast cancer. **a** Schematic diagram showing: the genomic loci of circNR3C2; the generation of circNR3C2 through back-splicing; the back-splice site confirmed by Sanger sequencing. **b** The convergent and divergent primers designed for detecting linear NR3C2 transcript and circNR3C2 respectively. **c** RT-PCR in cDNA and gDNA with the convergent and divergent primers showing the existence of circNR3C2 in breast cancer tissue and cells. GAPDH was used as a negative control. **d** RT-PCR with the divergent primers in total RNA showing the expression and stability of circNR3C2 in 5 breast cancer cell lines. N = control, R = RNase R treatment. **e** Subcellular localization of endogenous circNR3C2 in MCF-7 cells, presented via in situ hybridization with the FAM (fluorescein amidite)-labeled oligonucleotides probes. DAPI was used for staining the nucleus. Scale bar = 100 μm. **f** RT-qPCR showing the expression of circNR3C2 in luminal subtype and TNBC tissues. Luminal *N* = 30, TNBC *N* = 30. **g** Analysis of lymph node metastasis in 60 breast cancer samples with high (*N* = 27) or low (*N* = 33) circNR3C2 expression. The median expression value was used as cutoff. **h** Kaplan-Meier estimate showing the impact of circNR3C2 expression on the relapse-free survival of breast cancer patients (*N* = 60). The median expression value of circNR3C2 was used as cutoff

Located at chr4: 149356255–149,358,014, circNR3C2 with a full-length of 1760 nucleotides was detected as generated from back-splicing of the second exon of the NR3C2 (Nuclear Receptor Subfamily 3 Group C Member 2) gene (Fig. 6a). For checking the existence of circNR3C2, we designed convergent and divergent primers to amplify the linear exon sequence and the back-splice site of circNR3C2 (Fig. 6b). As indicated by RT-PCR, circNR3C2 was amplified only by divergent primers from cDNA but not gDNA (Fig. 6c), and the back-splice site was validated by Sanger sequencing in the meantime (Fig. 6a). Besides, total RNA extracted from five breast cancer cell lines was subjected to RT-PCR after treatment with RNase R, suggesting the stability of circNR3C2 and its significant underexpression in TNBC compared to luminal breast cancer cells (Fig. 6d). At last, fluorescence in situ hybridization (FISH) was conducted, demonstrating that circNR3C2 was primarily localized in the cytoplasm of MCF-7 cells while barely expressed in MDA-MB-231 cells (Fig. 6e, Supplementary Fig. 3).

As the existence, abundance, stability and localization of circNR3C2 had been identified, we next contrived to explore its association with clinical outcome in breast cancers. Quantitative PCR confirmed the crucially lower expression of circNR3C2 in TNBC against luminal breast cancer tissues (Fig. 6f), in line with the occurrence of lymph nodes metastasis (Fig. 6g). Furthermore, low expression of circNR3C2 was strongly correlated with poor prognosis due to the fact that patients with low circNR3C2 expression showed a remarkably shorter relapse-free survival and vice versa (Fig. 6h). Taken together, these results indicate the tumor-suppressive effect of circNR3C2.

### Overexpression of circNR3C2 upregulates HRD1 by sponging miR-513a-3p

**Fig. 7:**
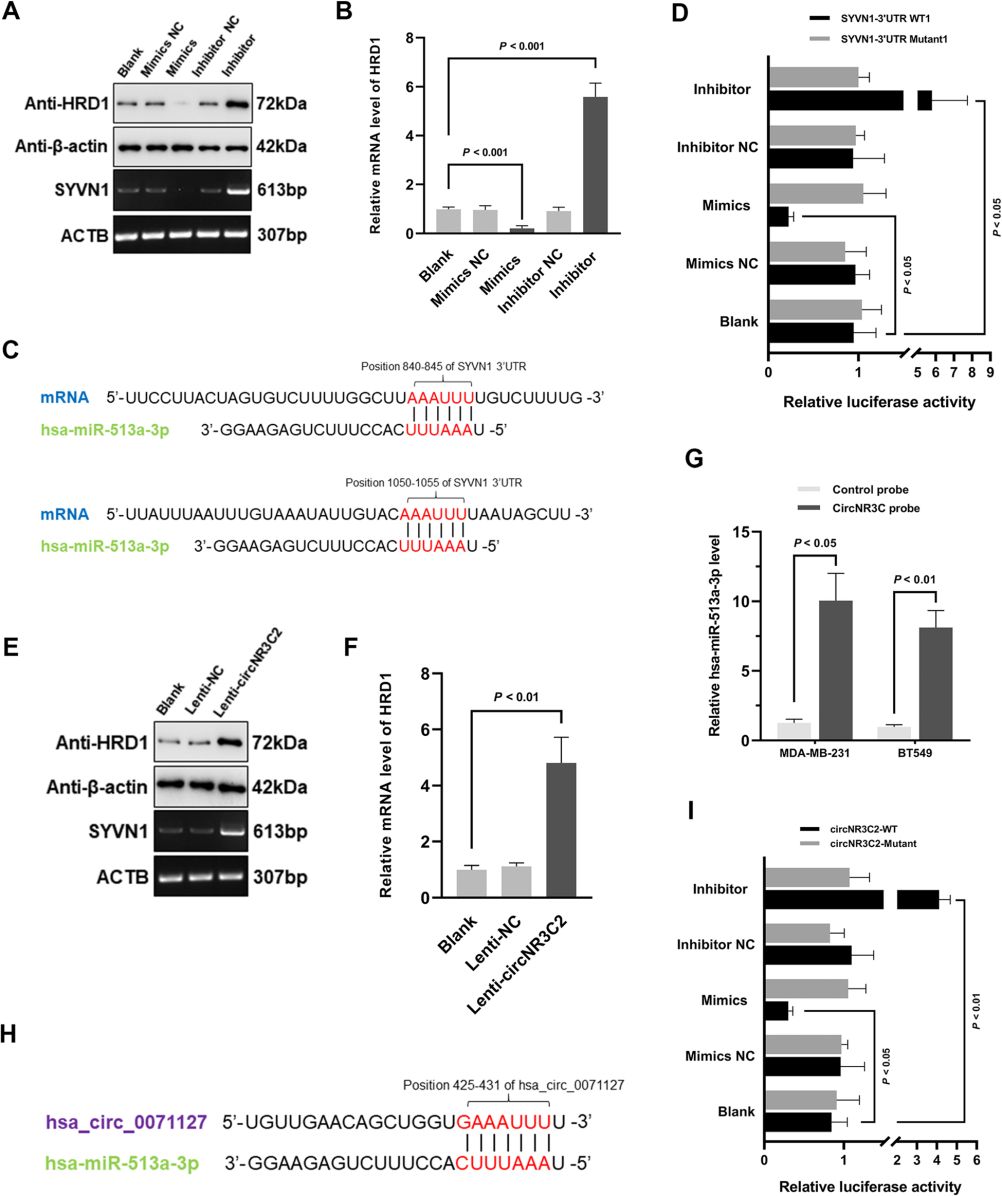
CircNR3C2 upregulates HRD1 via sponging hsa-miR-513a-3p in breast cancer. **a** Immunoblotting and RT-PCR showing the expression of HRD1 protein and mRNA in MDA-MB-231 cells either transfected with miR-513a-3p mimics or inhibitor, compared with corresponding negative control. β-actin was used as a loading control. **b** HRD1 mRNA level across the indicated groups of MDA-MB-231 cells, assessed with RT-qPCR. **c** Schematic representation of the predicted interacting sites of hsa-miR-513a-3p with HRD1 3’UTR. **d** Firefly luciferase activity in MDA-MB-231 cells cotransfected with reporter plasmids (GV272 vectors containing the 3’UTR of HRD1, harbouring the first wild-type miRNA binding site or its mutant) and the indicated oligonucleotides, normalized with renilla luciferase activity. **e**, **f** Immunoblotting and RT-PCR/qPCR showing the expression of HRD1 protein and mRNA in control and circNR3C2 overexpressing MDA-MB-231 cells with β-actin as an internal control. **g** RT-qPCR showing the relative level of hsa-miR-513a-3p pulled down with circNR3C2 in MDA-MB-231 and BT549 cells. **h** Schematic representation of the predicted interacting site of hsa-miR-513a-3p with circNR3C2. **i** Firefly luciferase activity in MDA-MB-231 cells cotransfected with reporter plasmids (GV272 vectors harbouring wild-type or mutated miRNA binding site of circNR3C2) and the indicated oligonucleotides, normalized with renilla luciferase activity. Data were represented as means ± S.D. of at least three independent experiments

CircNR3C2 and miR-513a-3p in combination were predicted to possibly regulate HRD1 expression in breast cancer as described above. Immunoblotting and RT-PCR showed that HRD1 protein and mRNA level was either downregulated or upregulated in MDA-MB-231 cells transfected with miR-513a-3p mimics or inhibitor (Fig. 7a), which was supported by RT-qPCR (Fig. 7b). To verify the binding of miR-513a-3p to HRD1 mRNA, a fragment of the 3’UTR containing predicted interacting site (Fig. 7c) or its mutant was amplified and cloned to luciferase reporter vector. As demonstrated by dual luciferase reporter assays, a significant reduction/increment of normalized firefly luciferase activity was detected in MDA-MB-231 cells transfected with miR-513a-3p mimics and inhibitor respectively, suggesting a bona fide interaction between the both (Fig. 7d and Supplementary Fig. 2A). Moreover, circNR3C2 overexpression was showed to upregulate the mRNA and protein level of HRD1 in MDA-MB-231 cells but without a decrease of miR-513a-3p (Fig. 7e, f and Supplementary Fig. 2B). The biotinylated DNA probe targeting circNR3C2 was synthesized for RNA pull-down assay, which demonstrated an interaction between circNR3C2 and miR-513a-3p in MDA-MB-231 and BT549 cells (Fig. 7g and Supplementary Fig. 2C). To further substantiate the sponging effect of circNR3C2 towards miR-513a-3p, we constructed luciferase reporter plasmids carrying the wild-type or mutant sequence of predicted interacting site in the same way (Fig. 7h). The similar alterations of luciferase activity among different groups were observed, indicating that circNR3C2 could also interact with miR-513a-3p as HRD1 mRNA (Fig. 7i). Collectively, these results suggest that the circNR3C2/miR-513a-3p/HRD1 axis truly exists in breast cancer cells.

### Overexpression of circNR3C2 reduces tumor growth and metastasis through Vimentin degradation

**Fig. 8:**
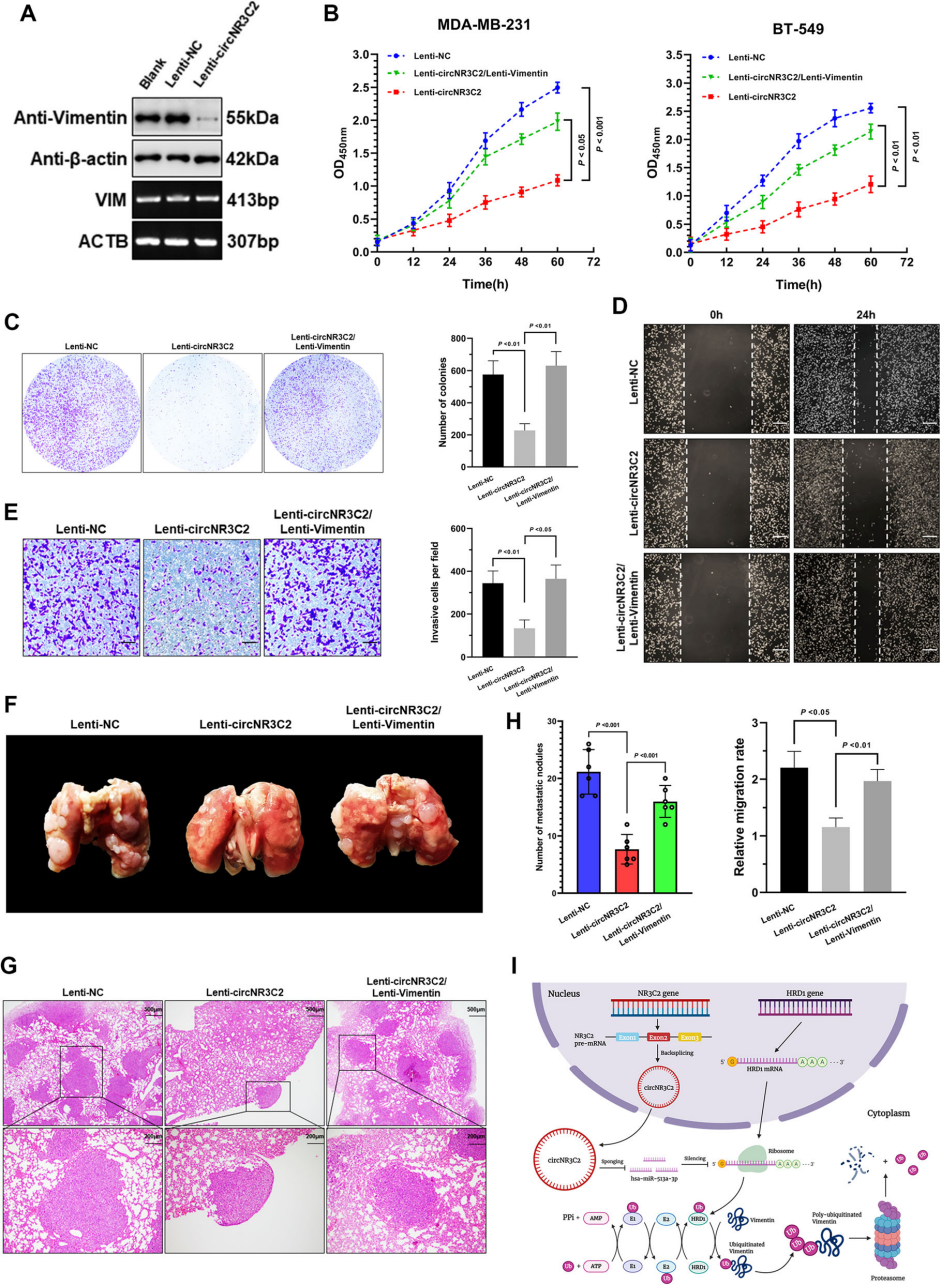
The circNR3C2/miR-513a-3p/HRD1 axis suppresses tumorigenesis and metastasis by downregulating Vimentin. **a** Immunoblotting and RT-PCR showing the expression of Vimentin protein and mRNA in control and circNR3C2-overexpressing MDA-MB-231 cells. **b** CCK-8 assay showing the optical density at 450 nm of MDA-MB-231 and BT549 cells stably overexpressing circNR3C2/Vimentin or singly circNR3C2, suggesting the number of living cells at the indicated timepoints. **c** Colonies formed by MDA-MB-231 cells stably overexpressing circNR3C2/Vimentin or singly circNR3C2 were stained with crystal violet solution, 15 days after seeding. **d** Cell migration indicated by the scratch width of MDA-MB-231 cells stably overexpressing circNR3C2/Vimentin or singly circNR3C2, 0 and 24 h after wound generation. Scale bar = 200 μm. **e** MDA-MB-231 cells stably overexpressing circNR3C2/Vimentin or singly circNR3C2, moving through matrigel in 12 h and adhering to the lower surface of the bottom membrane, were stained with crystal violet solution in transwell invasion assay. Scale bar = 100 μm. **f** Representative image of whole lungs harvested from BALB/c nude mice 7 weeks after intravenous injection with the indicated groups of MDA-MB-231 cells, showing the metastatic nodules. **g** Representative hematoxylin and eosin (H&E) staining showing the number of metastatic nodules of mouse lung tissues in Fig. 8 **f**. Scale bar = 500/200 μm. **h** Statistical analysis of the lung metastatic nodules in BALB/c nude mice 7 weeks after intravenous injection with the indicated groups of MDA-MB-231 cells. *N* = 6 per group. **i** Schematic diagram showing the regulatory mechanisms of the circNR3C2/miR-513a-3p/HRD1 axis on Vimentin protein stability in breast cancer. Data were represented as means ± S.D. of at least three independent experiments

Based on the above results, we assumed that overexpression of circNR3C2 should have an impact on Vimentin stability in TNBC cells. Indeed, MDA-MB-231 cells stably overexpressing circNR3C2 exhibited a significant lower expression of Vimentin at protein but not mRNA level compared to the wild type cells (Fig. 8a). Meanwhile, we verified the tumor-suppressive mechanism of circNR3C2 with the gain-of-function experiments in vitro and in vivo. Particularly, CCK-8 and colony formation assays showed that circNR3C2 overexpression inhibited the proliferation and reproductive capability to a great extent in TNBC cells (Fig. 8b, c), while in vivo tumor formation assay also indicated the suppressive role of circNR3C2 on tumorigenesis (Supplementary Fig. 4A-4C). On the other hand, parallel effects on tumor migration and invasion were demonstrated by wound healing and transwell assays (Fig. 8d, e). Notably, all of the suppressive effects of circNR3C2 on tumor progression could be reversed by re-expression of Vimentin. Finally, we established an in vivo metastasis model using MDA-MB-231 cells with different treatments intravenously injected to immunodeficient nude mice. Overexpression of circNR3C2 caused a significant reduction of lung metastasis along with metastatic pulmonary nodules, which was restored by Vimentin re-expression (Fig. 8f-8h). In summary, our findings suggest that circNR3C2 specially sponges miR-513a-3p, promoting HRD1-mediated degradation of Vimentin through ubiquitin-proteasome pathway thus ultimately suppressing tumor growth and metastasis of breast cancer (Fig. 8i).

## Discussion

In the landmark review titled *The Hallmarks of Cancer*, Douglas Hanahan and Robert Weinberg summarized six major acquired capabilities of cancer, one of which is the tissue invasion and metastasis [33]. While primary tumors are often cured by surgical resection and adjuvant therapy, metastatic cancer is nearly incurable or even untreatable because of the resistance of disseminated cancer cells to existing therapeutic agents, causing > 90% mortality from cancer [34]. Notably, in contrast to ER+ or HER2+ subtype that can be well-treated with endocrine or monoclonal antibody therapy, TNBC patients usually have a higher risk of relapse with distant metastasis even after receiving chemotherapy, owing to the lack of specific targeted therapies [35]. The invasion-metastasis cascade is a complex multi-step process comprised of sequential cell-biological events. A key step in the initiation of metastasis is the detachment, migration, and invasion of primary tumor cells, following the same paradigm of the process of EMT in embryonic morphogenesis. Therefore, the importance of EMT program in tumor progression has been established over the past two decades. It has been intensively studied that EMT-induced mesenchymal traits enable cancer cells to accomplish many steps of the invasion-metastasis cascade, from local invasion to intravasation, dissemination, extravasation, and colonization at distant sites [6, 7, 36]. With the activation of EMT transcription factors (SNAI1, TWIST1, ZEB1/2, etc.), genes encoding epithelial junction proteins like E-cadherin and claudin are transcriptionally downregulated, while the expression of mesenchymal genes such as N-cadherin, fibronectin, and Vimentin is upregulated [37].

As one of the major markers of mesenchymal state, Vimentin serves as an effector of EMT program. For instance, intermediate filaments are shown to switch from cytokeratin to Vimentin during EMT. Since the cytokeratin network anchored to desmosomes in epithelial cells is being deconstructed, the upregulation of Vimentin leads to reorganization of the basal network of intermediate filaments, facilitating cell motility and the formation of new membrane protrusions for extracellular matrix (ECM) degradation, finally resulting in cell migration and invasive behavior [38]. Besides being in the cytoplasm of mesenchymal cells where it functions to maintain the cytoarchitecture and tissue integrity, Vimentin has been shown to be a nuclear as well as extra cellular protein known to interact with a large number of binding partners and participate in intracellular signal transduction. It has been reported that Vimentin upregulation during EMT enhances tumorigenesis via targeting 14–3-3-mediated cell cycle control, and increases the migratory and invasive capacity of cancer cells by stabilizing scaffold protein SCRIB [9]. Furthermore, secreted extracellular Vimentin is associated with the spheroid formation of glioblastoma cancer stem cells [39], which is consistent with the acquisition of stemness in tumor cells undergoing EMT. Taken together, these findings suggest Vimentin as a potential biomarker and therapeutic target of breast invasive carcinoma and other aggressive cancers.

Among the ubiquitin-proteasome system (UPS) responsible for protein degradation in normal and pathological states, E3 ligases function to transfer ubiquitin activated by E1s and conjugated by E2s to substrates, regulating numerous cellular processes, including homeostasis, metabolism and cell cycle progression. Besides HECT (homologous to E6-AP carboxy terminus) E3 ligases, RING (really interesting new gene) and RING-related subgroup occupy the majority of E3 enzymes. Over 600 RING-type E3 ligases have been discovered, and many of them have been described to contribute to the pathogenesis of malignancy once dysregulated [40]. E3s recognize, interact with and ubiquitylate protein substrates in a spatiotemporally regulated manner, and both tumor-promotive and tumor-suppressive pathways are regulated by E3 ligases-mediated ubiquitination. Thus, it depends on the nature of their substrates that E3s function as whether oncogenes or tumor suppressors. Furthermore, even a single E3 ligase can have opposing functions as tumor promoter or suppressor simultaneously, depending on context or the type of cancer involved [41]. HRD1, an endoplasmic reticulum (ER)-localized RING-type E3 ligase, was demonstrated to have an anti-apoptosis effect in arthritis pathogenesis by inducing polyubiquitination-mediated proteasomal degradation of p53 [42]. However, accompanied with recent research revealing the inhibitory effect of HRD1 on TNBC tumorigenesis [43], from the perspective of our research, HRD1 plays a role of tumor suppressor due to its anti-metastasis activity targeting Vimentin in breast cancer. Although the binding sites of E3 ligases on target substrates are highly specific for ubiquitination, a single protein may be targeted by various E3 ligases and each E3 ligase could have multiple substrates. Equivalent in mechanism to existing evidence that RING-type E3 ligases TRIM56 and RNF208 perform tumor-suppressive functions through ubiquitination-dependent negative regulation of Vimentin [11, 12], we identify HRD1 as an emerging member of those RING E3 enzymes pivotal in cancer progression for the first time.

The dysregulation of E3s in cancer can be attributed to genetics, epigenetics, transcriptional and post-transcriptional/translational alterations. Accumulating evidence shows that circRNAs, a unique type of long non-coding RNAs (lncRNAs) with covalently closed loops formed through backsplicing, are abundant, stable and highly conserved in eukaryotes with gene-regulatory potency [44]. Recent studies have revealed that circRNAs are involved in the occurrence and progression of various cancers. In particular, an increasing number of circRNAs have been demonstrated to be involved in almost every pathological process of breast cancer, exerting their biological functions acting as miRNA and protein sponges, modulating parental gene transcription and protein translation [45]. MiRNAs can recognize mRNA targets at specific sequences termed miRNA response elements (MREs), ultimately silencing the expression of target genes at post-transcriptional level. Conversely, RNA transcripts including lncRNAs, circRNAs, mRNAs and transcribed pseudogenes that have the same MREs can competitively bind to miRNAs and block them from binding to their target sites, according to the ceRNA (competing endogenous RNA) theory [46]. After lncRNAs, circRNAs have become a new research focus among the ceRNA family. Given that HRD1 is significantly underexpressed in TNBC, we investigated whether there is a post-transcriptional regulating pathway by circRNAs, which account for the dysregulation of HRD1 in breast cancer. Based on the RNA sequencing data of circRNAs in TNBC and adjacent normal breast tissues [17], we predicted and identified circNR3C2 a bona fide governing factor of HRD1 expression by sponging miR-513a-3p, using bioinformatical methods and a series of functional verification experiments mentioned above. Although it has been revealed that circNR3C2 acts as a ceRNA to inhibit carcinogenesis and metastasis, the modulation of circNR3C2 biogenesis and the regulatory role of other differentially expressed circRNAs in TNBC still need to be further clarified.

## Conclusions

In conclusion, we elucidated a novel tumor-suppressive role of HRD1 via inducing polyubiquitination-mediated proteasomal degradation of Vimentin, which could be upregulated by circNR3C2 through sponging miR-513a-3p. Our study provides a functional annotation to one of the most dysregulated circRNAs in TNBC, suggesting HRD1 and circNR3C2 as the potential prognostic markers and therapeutic targets for patients with triple-negative breast cancer.

## Supplementary Information

**Additional file 1: Supplementary Figure 1.** HRD1 is correlated with longer relapse-free survival of breast cancer patients. Kaplan-Meier analysis showing the influence of HRD1 expression on relapse-free survival of overall breast cancer samples from multiple public microarray datasets collected and organized by *KM plotter*.

**Additional file 2: Supplementary Figure 2.** CircNR3C2 upregulates HRD1 via sponging miR-513a-3p in breast cancer. **a** Firefly luciferase activity in MDA-MB-231 cells cotransfected with reporter plasmids (GV272 vectors containing the 3’UTR of HRD1, harbouring the second wild-type miRNA binding site or its mutant) and the indicated oligonucleotides, normalized with renilla luciferase activity. **b** RT-qPCR showing the relative expression of miR-513a-3p in MDA-MB-231 and BT549 cells transfected with Lenti-NC or Lenti-circNR3C2. **c** RT-qPCR showing the relative level of circNR3C2 pulled down by control and circNR3C2-specific probe in MDA-MB-231 and BT549 cells. Data were represented as means ± S.D. of at least three independent experiments.

**Additional file 3: Supplementary Figure 3.** Fluorescence in situ hybridization of circNR3C2 in MDA-MB-231 cells. Subcellular localization of endogenous circNR3C2 in MDA-MB-231 cells, presented via in situ hybridization with the FAM-labeled oligonucleotides probes. DAPI was used for staining the nucleus. Scale bar = 100 μm.

**Additional file 4: Supplementary Figure 4.** CircNR3C2 overexpression inhibits in vivo tumorigenesis of TNBC via downregulating Vimentin. **a** Photograph showing the rough size of tumors generated from MDA-MB-231 cells stably overexpressing circNR3C2/Vimentin or singly circNR3C2 (*N* = 5 per group). **b** Line chart showing the tumor volume measured weekly, starting from two weeks after injection. **c** Weight of tumors isolated from nude mice, six weeks after injection.

**Additional file 5.**

## Data Availability

The public datasets analysed during the current study are available in the repositories listed below: *• Gene Expression Omnibus* GSE41313 https://www.ncbi.nlm.nih.gov/geo/query/acc.cgi?acc=GSE41313 GSE1456 https://www.ncbi.nlm.nih.gov/geo/query/acc.cgi?acc=GSE1456 GSE5460 https://www.ncbi.nlm.nih.gov/geo/query/acc.cgi?acc=GSE5460 GSE113230 https://www.ncbi.nlm.nih.gov/geo/query/acc.cgi?acc=GSE113230 *• The Cancer Genome Atlas* TCGA-BRCA https://portal.gdc.cancer.gov/projects/TCGA-BRCA *• Clinical Proteomic Tumor Analysis Consortium* Breast Invasive Carcinoma https://pdc.cancer.gov/pdc/ *• Kaplan-Meier plotter* Breast Cancer https://kmplot.com/analysis/index.php?p=service&cancer=breast

## Abbreviations

BafA1: Bafilomycin A1
CeRNA: Competing endogenous RNA
CHX: Cycloheximide
CircRNA: Circular RNA
CTPAC: Clinical Proteomic Tumor Analysis Consortium
EGF: Epidermal growth factor
EMT: Epithelial-mesenchymal transition
ER: Estrogen receptor
FFPE: Formalin-fixed, paraffin-embedded
FISH: Fluorescence in situ hybridization
HER2: Human epidermal growth factor receptor 2
HRD1: HMG-CoA reductase degradation protein 1
IHC: Immunohistochemistry
NR3C2: Nuclear receptor subfamily 3 group C member 2
PR: Progesterone receptor
RING: Really interesting new gene
TNBC: Triple-negative breast cancer
TCGA: The Cancer Genome Atlas
