# Supplementary figures and images for: CircNR3C2 promotes HRD1-mediated tumor-suppressive effect via sponging miR-513a-3p in triple-negative breast cancer
Source: Mol Cancer. 2021 Feb 2;20:25. doi: 10.1186/s12943-021-01321-x (PMC7851937; doi:10.1186/s12943-021-01321-x)

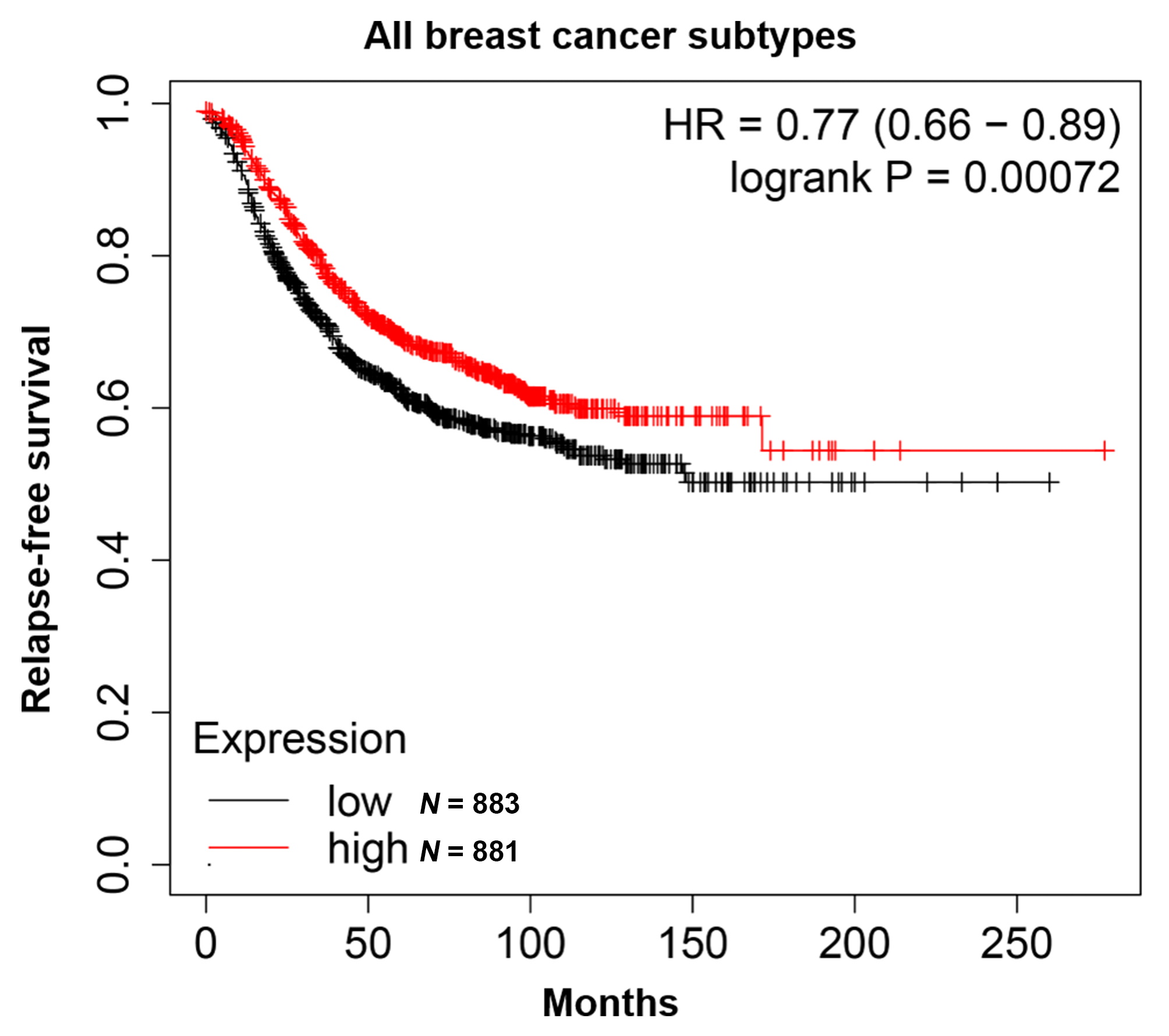

Supplement: Supplementary file 1 — Additional file 1: Supplementary Figure 1. HRD1 is correlated with longer relapse-free survival of breast cancer patients. Kaplan-Meier analysis showing the influence of HRD1 expression on relapse-free survival of overall breast cancer samples from multiple public microarray datasets collected and organized by KM plotter. [file 12943_2021_1321_MOESM1_ESM.tif]

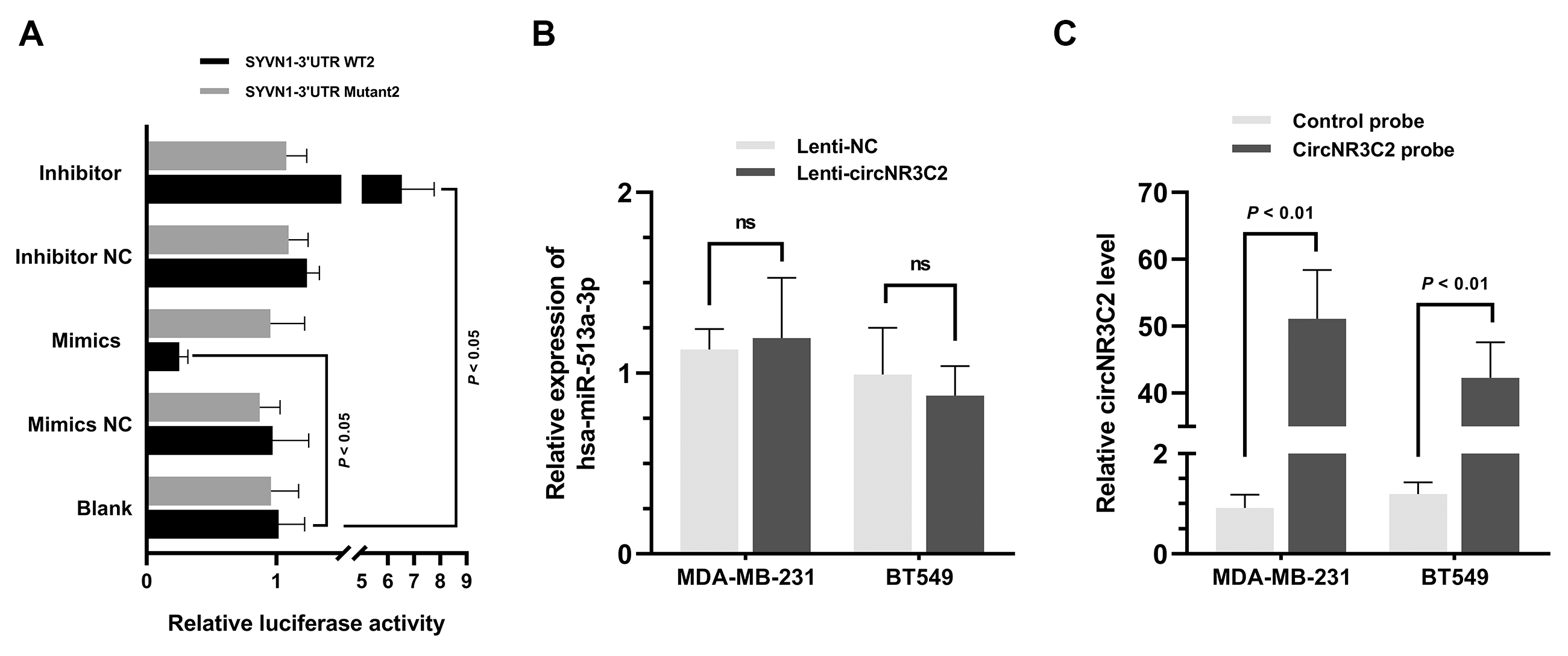

Supplement: Supplementary file 2 — Additional file 2: Supplementary Figure 2. CircNR3C2 upregulates HRD1 via sponging miR-513a-3p in breast cancer. a Firefly luciferase activity in MDA-MB-231 cells cotransfected with reporter plasmids (GV272 vectors containing the 3’UTR of HRD1, harbouring the second wild-type miRNA binding site or its mutant) and the indicated oligonucleotides, normalized with renilla luciferase activity. b RT-qPCR showing the relative expression of miR-513a-3p in MDA-MB-231 and BT549 cells transfected with Lenti-NC or Lenti-circNR3C2. c RT-qPCR showing the relative level of circNR3C2 pulled down by control and circNR3C2-specific probe in MDA-MB-231 and BT549 cells. Data were represented as means ± S.D. of at least three independent experiments. [file 12943_2021_1321_MOESM2_ESM.tif]

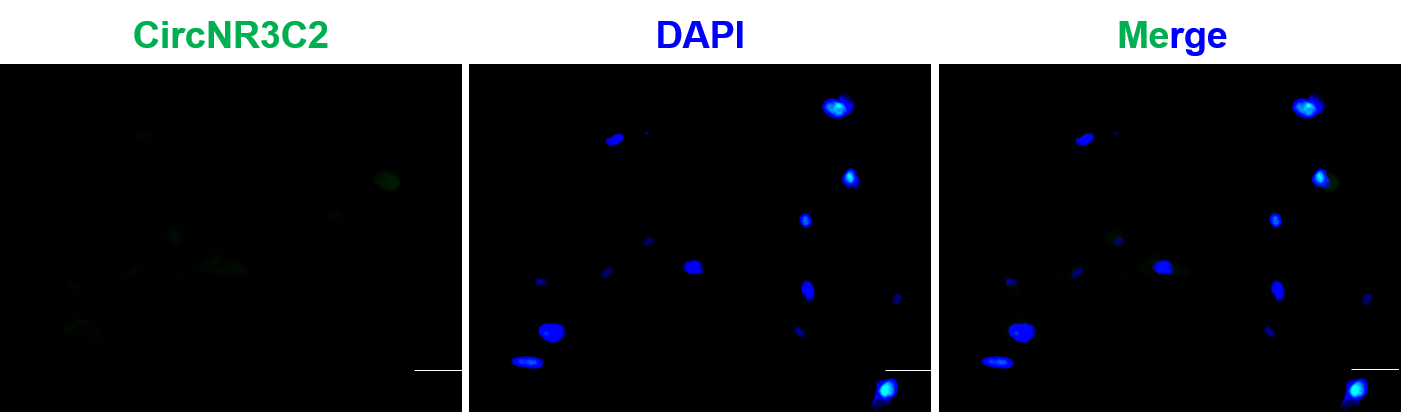

Supplement: Supplementary file 3 — Additional file 3: Supplementary Figure 3. Fluorescence in situ hybridization of circNR3C2 in MDA-MB-231 cells. Subcellular localization of endogenous circNR3C2 in MDA-MB-231 cells, presented via in situ hybridization with the FAM-labeled oligonucleotides probes. DAPI was used for staining the nucleus. Scale bar = 100 μm. [file 12943_2021_1321_MOESM3_ESM.png]

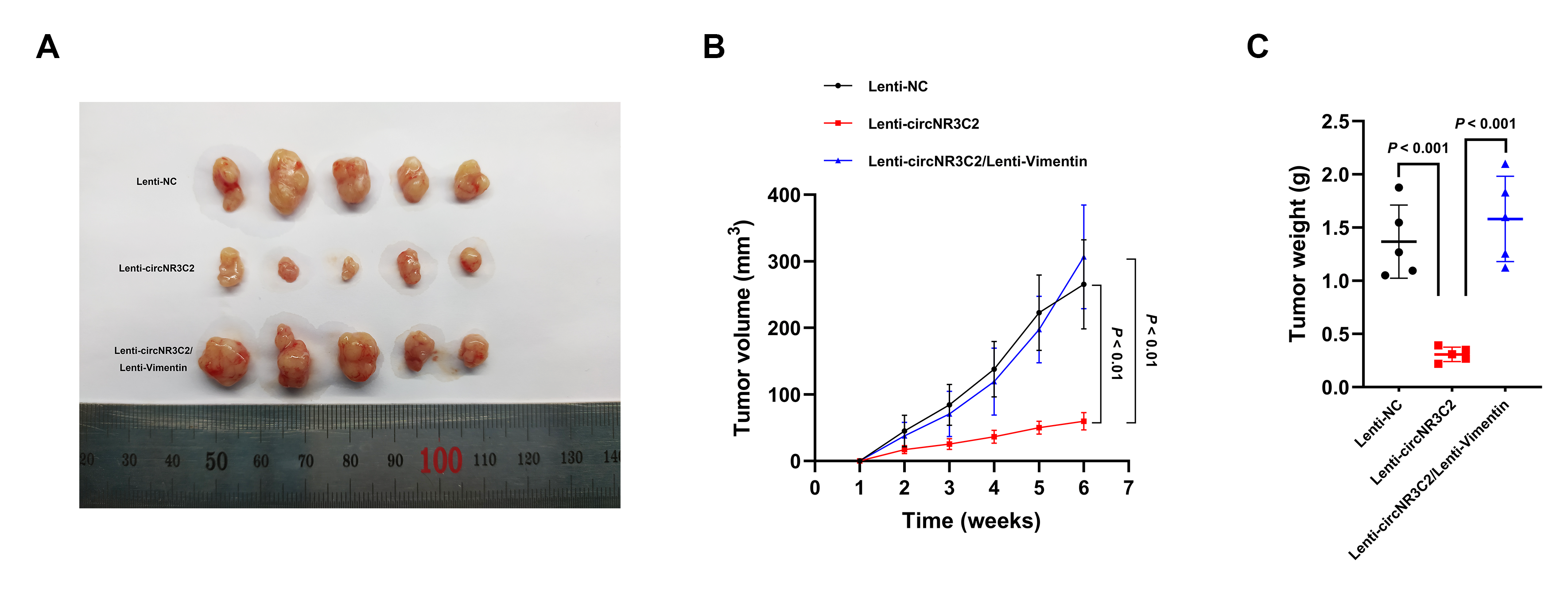

Supplement: Supplementary file 4 — Additional file 4: Supplementary Figure 4. CircNR3C2 overexpression inhibits in vivo tumorigenesis of TNBC via downregulating Vimentin. a Photograph showing the rough size of tumors generated from MDA-MB-231 cells stably overexpressing circNR3C2/Vimentin or singly circNR3C2 (N = 5 per group). b Line chart showing the tumor volume measured weekly, starting from two weeks after injection. c Weight of tumors isolated from nude mice, six weeks after injection. [file 12943_2021_1321_MOESM4_ESM.png]
