# Supplementary material for: CircNR3C2 promotes HRD1-mediated tumor-suppressive effect via sponging miR-513a-3p in triple-negative breast cancer
Source: Mol Cancer. 2021 Feb 2;20:25. doi: 10.1186/s12943-021-01321-x (PMC7851937; doi:10.1186/s12943-021-01321-x)
Supplement: Supplementary file 5 — Additional file 5. [file 12943_2021_1321_MOESM5_ESM.docx]

1. **Primer sequences for RT-PCR (5’-3’)**

**SYVN1 (HRD1)**

Forward: TGCGTAACATCCACACACTG

Reverse: AGGCTAAACCTTCTGCCTTCA

**VIM (Vimentin)**

Forward: AATGCTTCTCTGGCACGTCT

Reverse: TCTTCCATCTCACGCATCTG

**ACTB (β-actin)**

Forward: AATGCTTCTCTGGCACGTCT

Reverse: TCTTCCATCTCACGCATCTG

**CircNR3C2 (hsa_circ_0071127)**

Forward (convergent): CAGTTTTCCAGTGCACAGCC

Reverse (convergent): ACCTTTCTCCTGCGTGTCTG

Forward (divergent): TCATGGAAATCACACGGCGA

Reverse (divergent): TCATCGGTCCTCTCTGTAGGT

**GAPDH**

Forward (convergent): CCATGGGGAAGGTGAAGGTC

Reverse (convergent): GACTCCACGACGTACTCAGC

Forward (divergent): GTGCTCAACCAGTTGGCACC

Reverse (divergent): AGCCTCGCTCCACCTGACTT

1. **Primer sequences for RT-qPCR (5’-3’)**

**SYVN1 (HRD1)**

Forward: CCCTAGGCCAAGTGGAAAGAG

Reverse: GGGCTTCTCAGAGGCTAAACC

**VIM (Vimentin)**

Forward: TCCGCACATTCGAGCAAAGA

Reverse: TGATTCAAGTCTCAGCGGGC

**ACTB (β-actin)**

Forward: CTCACCATGGATGATGATATCGC

Reverse: CCACATAGGAATCCTTCTGACC

**CircNR3C2 (hsa_circ_0071127)**

Forward (divergent): TCATGGAAATCACACGGCGA

Reverse (divergent): GCTCCACAGCCTGAGAAACT

**Hsa-miR-513a-3p**

Forward: ACACTCCAGCTGGGUAAAUUUCACCUUUCUG

Reverse: CTCAACTGGTGTCGTGGAGTCGGCAATTCAGTTGAGCCTTCTCA

1. **Sequences cloned to luciferase reporter vector (5’-3’)**

**HRD1 3’UTR wild-type 1**

TGACCACTGCAATCCCAGCGCCCAAGGAAGGCCACTTCTCAACTGGCAGAACTTCTGAAGTTTAGAATTGGAATTACTTCCTTACTAGTGTCTTTTGGCTTAAATTTTGTCTTTTGAAGTTGAATGCTTAATCCCGGGAAAGAGGAACAGGAGTGCCAGACTCCTGGTCTTTCCAGTTTAGAAAAGGCTCTGTGCCAAGGAGGGACC

**HRD1 3’UTR mutant 1**

TGACCACTGCAATCCCAGCGCCCAAGGAAGGCCACTTCTCAACTGGCAGAACTTCTGAAGTTTAGAATTGGAATTACTTCCTTACTAGTGTCTTTTGGCTTCCCGGGTGTCTTTTGAAGTTGAATGCTTAATCCCGGGAAAGAGGAACAGGAGTGCCAGACTCCTGGTCTTTCCAGTTTAGAAAAGGCTCTGTGCCAAGGAGGGACC

**HRD1 3’UTR wild-type 2**

GGAGCTGGGACCTGCCTGCCCCTGTCTTTTCCCCTTGGTTTTGTGTTACAAGAGTTGTTGGAGACAGTTTCAGATGATTATTTAATTTGTAAATATTGTACAAATTTTAATAGCTTAAATTGTATATACAGCCAAATAAAAACTTGCATTAACAA

**HRD1 3’UTR mutant 2**

GGAGCTGGGACCTGCCTGCCCCTGTCTTTTCCCCTTGGTTTTGTGTTACAAGAGTTGTTGGAGACAGTTTCAGATGATTATTTAATTTGTAAATATTGTACCCCGGGTAATAGCTTAAATTGTATATACAGCCAAATAAAAACTTGCATTAACAA

**CircNR3C2 wild-type**

ATATGGATTCTGTAAGAGATGCTGACTATTCCTATGAGCAGCAGAACCAACAAGGAAGCATGAGTCCAGCTAAGATTTATCAGAATGTTGAACAGCTGGTGAAATTTTACAAAGGAAATGGCCATCGTCCTTCCACTCTAAGTTGTGTGAACACGCCCTTGAGATCATTTATGTCTGACTCTGGGAGCTCCGTGAATGGTGGCGTCA

**CircNR3C2 mutant**

ATATGGATTCTGTAAGAGATGCTGACTATTCCTATGAGCAGCAGAACCAACAAGGAAGCATGAGTCCAGCTAAGATTTATCAGAATGTTGAACAGCTGGTTCCCGGGTACAAAGGAAATGGCCATCGTCCTTCCACTCTAAGTTGTGTGAACACGCCCTTGAGATCATTTATGTCTGACTCTGGGAGCTCCGTGAATGGTGGCGTCA

1. **Sequences of FISH probes (5’-3’)**

**CircNR3C2 (hsa_circ_0071127) probe**

ATCGCT+TGATACATTTTCTGGA+ATGGTCTCCA+TCGCTTGATACATT+TTCTGGT+AGCCTTTGGTCTCCA+TCGCTTG

**Positive control (18S rRNA) probe**

CTGCCTTCCTTGGATGTGGTAGCCGTTTC

**Negative control probe**

TGCTTTGCACGGTAACGCCTGTTTT
